# Lateral hypothalamic leptin receptor neurons drive hunger-gated food-seeking and consummatory behaviours in male mice

**DOI:** 10.1038/s41467-023-37044-4

**Published:** 2023-03-17

**Authors:** Young Hee Lee, Yu-Been Kim, Kyu Sik Kim, Mirae Jang, Ha Young Song, Sang-Ho Jung, Dong-Soo Ha, Joon Seok Park, Jaegeon Lee, Kyung Min Kim, Deok-Hyeon Cheon, Inhyeok Baek, Min-Gi Shin, Eun Jeong Lee, Sang Jeong Kim, Hyung Jin Choi

**Affiliations:** 1 Department of Biomedical Sciences, Seoul National University College of Medicine, 103 Daehak-ro, Jongno-gu Seoul, 03080 Republic of Korea; 2 Department of Anatomy and Cell Biology, Seoul National University College of Medicine, 103 Daehak-ro, Jongno-gu Seoul, 03080 Republic of Korea; 3 Department of Physiology, Seoul National University College of Medicine, 103 Daehak-ro, Jongno-gu Seoul, 03080 Republic of Korea; 4 Department of Brain Science, Ajou University School of Medicine, Suwon, 16499 Republic of Korea; 5 Neuroscience Research Institute, Seoul National University College of Medicine, 103 Daehak-ro, Jongno-gu Seoul, 03080 Republic of Korea; 6 Wide River Institute of Immunology, Seoul National University, 101 Dabyeonbat-gil, Hwachon-myeon Gangwon-do, 25159 Republic of Korea

**Keywords:** Hypothalamus, Reward

## Abstract

For survival, it is crucial for eating behaviours to be sequenced through two distinct seeking and consummatory phases. Heterogeneous lateral hypothalamus (LH) neurons are known to regulate motivated behaviours, yet which subpopulation drives food seeking and consummatory behaviours have not been fully addressed. Here, in male mice, fibre photometry recordings demonstrated that LH leptin receptor (LepR) neurons are correlated explicitly in both voluntary seeking and consummatory behaviours. Further, micro-endoscope recording of the LH^LepR^ neurons demonstrated that one subpopulation is time-locked to seeking behaviours and the other subpopulation time-locked to consummatory behaviours. Seeking or consummatory phase specific paradigm revealed that activation of LH^LepR^ neurons promotes seeking or consummatory behaviours and inhibition of LH^LepR^ neurons reduces consummatory behaviours. The activity of LH^LepR^ neurons was increased via Neuropeptide Y (NPY) which acted as a tonic permissive gate signal. Our results identify neural populations that mediate seeking and consummatory behaviours and may lead to therapeutic targets for maladaptive food seeking and consummatory behaviours.

## Abstract (web summary)

Eating behviours consist of seeking and consummatory phases. Here, authors show that two distinct lateral hypothalamic leptin receptor neurons orchestrate seeking and consummatory phases of eating behaviour via hunger signal, Neuropeptide Y.

## Introduction

Eating consists of various motivated behaviours. Each such behaviour is regulated by distinct drives modulated by external information and internal state information^1–3^. Furthermore, these eating behaviours are multi-phase behaviours which initiate with appetitive phase (seeking behaviours), that sequentially leads to a consummatory phase (when animal is proximate to the food, manipulation of food by biting, chewing then finishing with intake of food occurs)^4,5^. Since seeking and consummatory behaviours have distinct characteristics in aspects of motivational state and behavioural decision, it is physiologically crucial for two distinct functional populations to guide each behaviour. Similarly, regarding other context motivated behaviours (mating or aggression), previous studies have shown that seeking and consummatory behaviours are regulated by distinct neural populations^6–8^. Although several studies have investigated food seeking and consummatory neurons^9,10^, the identity of these two distinct neuronal populations is yet to be clarified.

Several studies have highlighted the lateral hypothalamus gamma aminobutyric acid (LH^GABA^) neurons to be heterogenous^9,11–13^ populations driving various motivated behaviours such as food consumption^9,14^, chewing objects^15,16^ exploring novel environments^17,18^, thermoregulation^19^ and social interaction^20^. In addition, even within the eating context, it has been suggested that there are two distinct LH^GABA^ neuron populations that encode seeking and consummatory behaviours^9,10^. These findings suggest that there could be two distinct eating specific subpopulations of LH^GABA^ neurons which are responsible for appetitive and consummatory behaviours. To elucidate which subpopulations in LH^GABA^ neurons exclusively contribute to seeking and/or consummatory behaviours, several studies have been dedicated to identifying subpopulations and neural circuits^10,16,17,20^. LH leptin receptor expressing (LH^LepR^) neurons are subpopulation of LH^GABA^ neurons and has been reported to be associated with eating^10,16,21–23^. However, the role of LH^LepR^ neurons is controversial; no effect on eating^10,24^, decreased eating^16^, decreased eating after leptin treatment in the LH^22^, and increased eating by LH^LepR^–Ventrolateral Periaqueductal Grey (vlPAG) circuit^23^.

Employing in vivo calcium imaging and phase specific behavioural tasks, we identified two distinct LH^LepR^ neural populations that are separately activated during seeking and consummatory behaviours, respectively. Further, neural activation results clearly demonstrated that LH^LepR^ neurons are sufficient for driving seeking behaviours and consummatory behaviours. Also, neural inhibition results clearly showed that LH^LepR^ neurons are necessary for driving consummatory behaviours.

Given that Agouti-related peptide/neuropeptide Y(AgRP/NPY) neurons deliver food-need information to downstream neurons (including the LH) via NPY^25^, we hypothesized that NPY neurotransmitters regulate LH^LepR^ neural activity. The present study showed that NPY is sufficient to increase LH^LepR^ neural activity through a disinhibition mechanism. Collectively, these data highlight the orchestration of eating phases within the LH circuitry and how these circuits are regulated by hunger signals.

## Results

### Overview of multiphasic experimental paradigms

To investigate seeking and consummatory behaviours, we developed phase specific tests to dissect two phases via temporal distinctions. behaviours, we developed seeking phase specific tests, which minimised consummatory behaviours (manipulating, licking, biting, chewing, and swallowing). To exclusively measure consummatory behaviours, we developed consummatory phase specific tests, which minimised seeking behaviours (searching and digging) (Supplementary Fig. 9a).

### LH^LepR^ neurons are the food-specific subpopulation of LH^GABA^ neurons

To investigate the heterogeneous LH^GABA^ neurons and test if LH^LepR^ neurons are part of food-specific LH^GABA^ subpopulation, we first investigated the anatomical distribution of LH^LepR^ neurons via whole-LH three-dimensional (3D) tissue clearing (Supplementary Movie 1) and 2D histological mapping using LepR-tdTomato mice (Supplementary Fig. 1a–k). As a result, LH^LepR^ neurons were mainly distributed in the middle region (−1.5 mm from bregma).

**Fig. 1:**
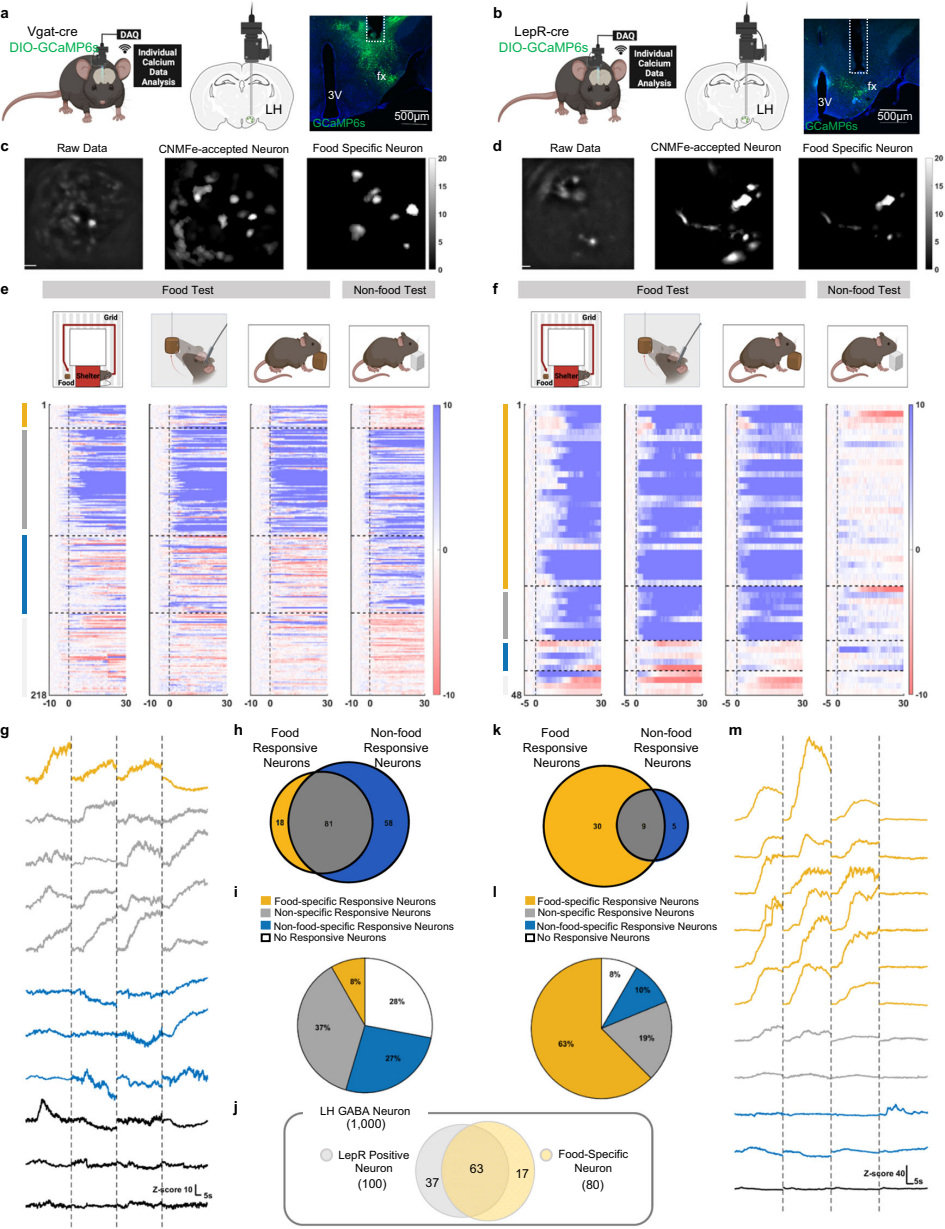
LH^LepR^ neurons are the food-specific subpopulation of LH^GABA^ neurons. **a**, **b** Schematic of micro-endoscopic calcium imaging (left, middle), and image of GCaMP6s expression (right) in the LH from Vgat-cre (**a**) and LepR-cre mice (**b**). The experiment was repeated 6 times (**a**) or 4 times (**b**) independently with similar results. fx, fornix; 3 V, the 3rd ventricle. **c**, **d** Spatial map of raw data (left), accepted cells using CNMFe (middle), and cells that only respond to food-related behaviour (right) from LH^GABA^ neurons (**c**) and LH^LepR^ neurons (**d**). Cells are coloured according to the maximum Z-score. Scale bar: 50 μm. **e**, **f** Schematic of the multi-phase test 2, consummatory behaviour test 1, consummatory behaviour test 2 (food and non-food) (top). Heatmap depicting calcium signals aligned to the onset of feeding behaviours (running to food, rearing to food, contact with food, contact with edible object) (below). Four populations are discriminated: food-specific responsive (yellow), non-specific responsive (grey), non-food-specific responsive (blue), and non-responsive (white) cells. (LH^GABA^ neurons 218 cells, 6 mice (**e**), LH^LepR^ neurons 48 cells, 4 mice (**f**)). **g**, **m** Representative traces of four populations from LH^GABA^ neurons (**g**) and LH^LepR^ neurons (**m**). The dotted line separates each behavioural experiment. **h**, **k** Venn diagram of food responsive and non-food responsive neurons. Percentage of food-responsive neurons are as follows (LH^GABA^ neurons 8% (18/218 cells) (**h**), LH^LepR^ neurons 63% (30/48 cells) (**k**)). **i**, **l** Proportion of food-specific responsive (yellow), non-specific responsive (grey), non-food-specific responsive (blue), and non-responsive (white) cells from LH^GABA^ neurons (**i**) and LH^LepR^ neurons (**l**). **j** Venn diagram simulating the number of LH^LepR^ positive (yellow) and food-specific (grey) neurons when the total number of LH ^GABA^ neurons is simulated as 1000. Source data are provided as a Source data file. The schematics in **a**, **b**, **e** and **f** were created using BioRender.

According to our mapping results, vesicular GABA transporter (Vgat)-cre and LepR-cre mice were injected with cre-dependent adeno-associated virus (AAV) carrying GCaMP6s and implanted a gradient index (GRIN) lens in the middle LH (Fig. 1a, b). Using micro-endoscopic imaging of calcium dynamics, we analysed three eating behaviours in fasted mice; running toward expected food (seeking behaviour, Fig. 1e, f left in the food test), approach toward proximate food (consummatory behaviour, Fig. 1e, f middle in food test) and chewing the proximate food (consummatory behaviour, Fig. 1e, f right in the food test). We first measured individual LH^GABA^ neural activity (Fig. 1c) during these tests compared to non-food behavioural test (chewing behaviour towards inedible Lego brick, Fig. 1e, f non-food test). We defined neurons as food-specific responsive (yellow) when they were activated during all three eating behavioural tests and not activated during a non-food behavioural test. Non-food-specific responsive neurons (blue), non-specific responsive neurons (grey), and no responsive neurons (white) were defined based on neural activity patterns during the tests (see ‘Methods’).

Among LH^GABA^ neurons, most neurons (64%) were activated in non-food behavioural tests (Fig. 1e, g–i, grey and blue panels). Instead, only a small subpopulation of neurons (8%) was food-specific responsive neurons (activated only in eating behaviour-related tests) (Fig. 1e, g–i, yellow panel), suggesting that only a small food-specific subpopulation exists within the vast total population of LH^GABA^ neurons. Of note, when LH^LepR^cre mice conducted the same experiments (Fig. 1f), most LH^LepR^ neurons (63%) were food-specific responsive (Fig. 1f, k–m, yellow panel).

Based on the previous single-cell RNA sequencing data for the LH^26^, LH^LepR^ neurons are mostly GABAergic (Supplementary Fig. 1l), consistent with previous results^21,23^. Further, LH^LepR^ neurons constitute only 4% of LH^GABA^ neurons (VGAT positive cells) (Supplementary Fig. 1m). A previous study has reported that LH^LepR^ neurons constitute less than 20% of LH^GABA^ neurons^21^. Although LH^LepR^ neurons represented only a minor portion (4–20%) of LH^GABA^ neurons (Supplementary Fig. 1m), our results indicate that most (79%; 63/80) of food-specific responsive LH^GABA^ neurons are LH^LepR^ neurons (Fig. 1j, Supplementary Fig. 2k). Furthermore, LH^LepR^ neurons were not activated to non-food investigation (Supplementary Fig. 2f–j). Compared to the robust response to food, only a minor response was observed to water (Supplementary Fig. 2a–e). These results suggest that LH^LepR^ neurons are food-specific population among LH^GABA^ neurons.

### LH^LepR^ neurons are activated during seeking and consummatory behaviours

**Fig. 2:**
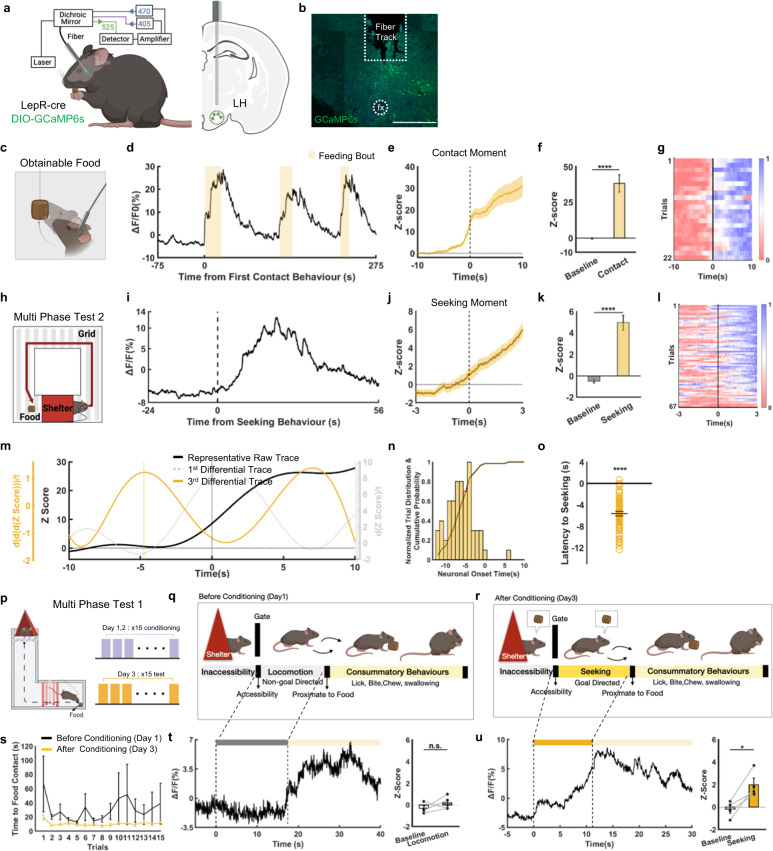
Activity of LH^LepR^ neurons is time-locked to seeking and consummatory behaviours. **a** Schematic of virus injection/fibre insertion for fibre photometry in LH from LepR-cre mice. **b** A representative image validates GCaMP6s expression in LepR neurons and optical fibre tract above the LH. Scale bar: 500 µm. The experiment was repeated 5 times independently with similar results. fx, fonix. **c** Schematic of the consummatory behaviour test 1 (obtainable). **d** Representative calcium traces from LH^LepR^ neurons. Yellow shaded box: from the moment of food contact to the end of food consumption. **e** Average Z-score from LH^LepR^ calcium response aligned to contact with food (5 mice, 22 trials). **f** Quantification of Z-score in calcium signal change from (**e**). Comparison between baseline (−8 to −7 s) and after contact (9 to 10 s). **g** Heatmap depicting normalised LH^LepR^ neural activity aligned to the moment of contact with food. **h** Schematic of the multi-phase test 2. **i**, **j** Representative calcium traces (**i**) and average Z-score (**j**) from LH^LepR^ neural calcium signal response aligned to voluntary seeking initiation (4 mice, 67 trials). **k** Quantification of Z-score in calcium signal change from (**j**). Comparison between baseline (−8 to −7 s) and after voluntary seeking behaviour (2 to 3 s). **l** Heatmap depicting normalised LH^LepR^ calcium signal aligned to voluntary seeking behaviour. **m** Representative neural onset of LH^LepR^ calcium signal aligned to the onset of voluntary seeking behaviour. Yellow line near –5 s is the neuronal onset timing. **n**, **o** Cumulative probability distribution (**n**) and histogram (**o**) of LH^LepR^ neural onset (4 mice, 66 trials). Neural onset occurred at −5.623 ± 0.418 s. **p** Schematic and schedule of the multi-phase test 1. **q**, **r** Dynamic feeding phase before conditioning (**q**) and after conditioning (**r**). **s** Time from food accessibility to food contact before and after conditioning (*n* = 4 mice). **t**, **u** Representative calcium signal of LH^LepR^ neurons aligned to food accessibility (left) and quantification of Z-score in calcium signal change (right) before (**t**) and after (**u**) conditioning. Comparison between baseline (−2 to −1 s) and after locomotion or seeking behaviour (1 to 2 s). Two-sided paired t-test; n.s., *p* > 0.05 (**t**), *p = 0.02 (**u**). Data are mean ± s.e.m. See Supplementary Table 1 for statistics. Source data are provided as a Source data file. The schematics in **a**, **c**, **h**, **p**, **q** and **r** were created using BioRender.

Next, to investigate temporal dynamics of LH^LepR^ neural activity during eating behaviour, neural activity was measured using fibre photometry at the population level (Fig. 2a, b). LH^LepR^ neural activity significantly increased at each eating bout with time-locked temporal dynamics in fasted mice (Fig. 2c–g, Supplementary Fig. 2f–j, Supplementary Movie 2). Interestingly, LH^LepR^ neural activity increased even before physical contact with food, implying that LH^LepR^ neurons may also be involved in seeking behaviours.

To precisely measure the temporal onset of LH^LepR^ neural activity with regard to voluntary behavioural onset, fasted mice were conditioned with random probability of electrical shock in the maze. Therefore, the mice hesitated before initiating seeking behaviours from the shelter. When the drive for food was higher than the fear of an electric shock, mice made the voluntary decision to initiate seeking. As expected, LH^LepR^ neural activity began to increase significantly before the mice initiated voluntary seeking (Fig. 2h–l, Supplementary Movie 2).

To precisely analyse the LH^LepR^ neural activity onset, we calculated the derivatives from polynomial regression traces from calcium activity traces^27,28^. By calculating the time point when the 3rd derivative reaches its maximum value, we calculated the neuronal onset when the neuronal activity begins to increase. As a result, LH^LepR^ neural activity onset significantly preceded the onset of seeking by an average of approximately 6 s (Fig. 2m–o). Additional tests revealed that LH^LepR^ neural activity decreased when mice voluntarily terminated both seeking or consummatory behaviours (Supplementary Fig. 3a–j). These results provide temporal causality evidence that LH^LepR^ neurons are the cause and drive for voluntary seeking behaviours, not the consequence of seeking behaviour (Supplementary Fig. 3a–j, Supplementary Movie 2), suggesting that LH^LepR^ neural activity is associated with voluntary behaviours.

To dissect seeking and consummatory phase, we developed a multi-phase test to provide sufficient temporal distinction between seeking and consummatory behaviours (Fig. 2p, Supplementary Movie 2). In the L-shaped chamber, fasted mice sequentially explored an empty corridor and arrived proximate to food. Before conditioning, mice explored the whole maze since mice were not aware of the food location (non-goal-directed locomotion, Fig. 2q). LH^LepR^ neural activity did not increase during this non-goal-directed locomotion (Fig. 2t). LH^LepR^ neural activity significantly started to increase when mice conducted consummatory behaviours at the end of the corridor. However, after conditioning (Fig. 2r), the mice moved directly to the food at the end of the corridor (goal-directed seeking; significantly shorter time from accessibility to food contact) (Fig. 2s). When compared with the neural activity results before conditioning, LH^LepR^ neural activity started to increase significantly when the mice initiated seeking, and there was an additional activity increase in the consummatory phase (Fig. 2u).

### Two distinct subpopulations of LH^LepR^ neurons individually encode seeking and consummatory behaviours

**Fig. 3:**
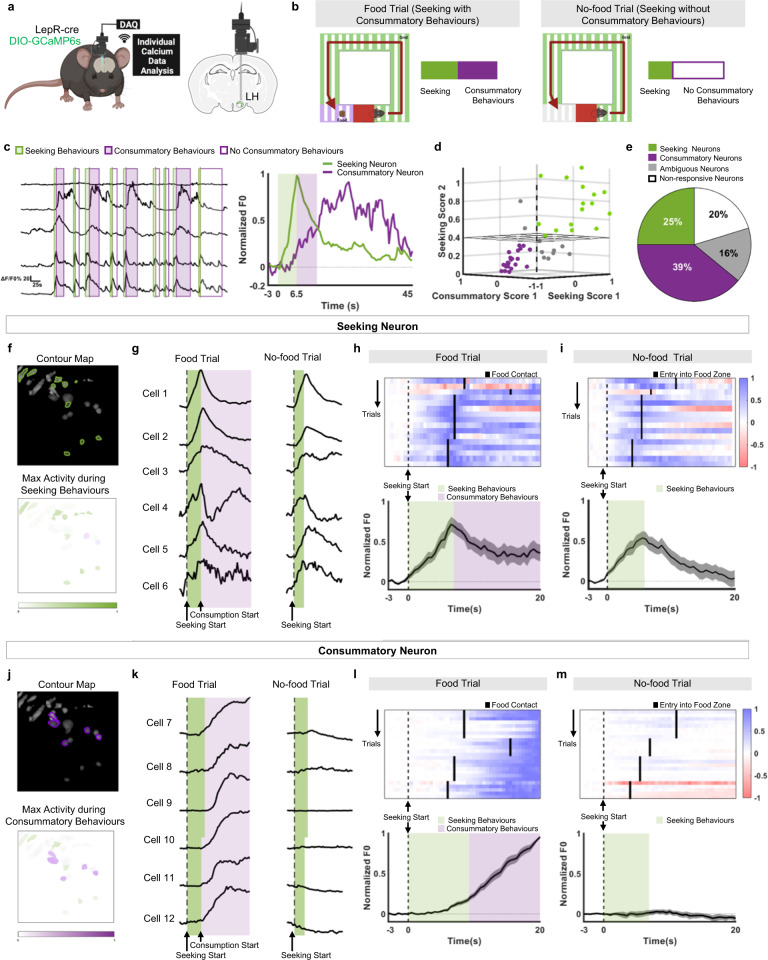
Two distinct populations of LH^LepR^ neurons encode seeking and consummatory behaviours. **a** Schematic of virus injection/GRIN lens insertion for micro-endoscopic calcium imaging in the LH from LepR-cre mice. **b** Schematic of the multi-phase test 2. Seeking with consummatory behaviours, in the presence of food (left). Seeking without consummatory behaviours, in the absence of food (right). **c** Representative single-cell traces of LH^LepR^ neurons within several trials (left) and one trial (right). Green shaded box indicated seeking behaviours and purple shaded box indicated consummatory behaviours. **d** Three-dimensional scored plot of LH^LepR^ neurons. **e** Proportion of cell populations. **f**, **j** Representative contour map of seeking (**f**, green) and consummatory (**j**, purple) neurons of an accepted cell (top). The degree of colour brightness represents the cell activity degree (max Z-score) (bottom). **g**, **k** Representative single-cell traces of LH^LepR^ neurons of seeking (**g**) and consummatory (**k**) neurons during food and no-food trials. **h**, **i**, **l**, **m** Heatmap depicting the calcium signals (top) and average Z-scores (bottom) of seeking neurons (**h**, **i**) or consummatory neurons (**l**, **m**). The magnitude of the calcium signals corresponds to its colour density. (4 mice, 15 cells (**h**, **i**), 4 mice, 25 cells (**l**, **m**)). Data are mean ± s.e.m. See Supplementary Table 1 for statistics. Source data are provided as a Source data file. The schematics in **a** and **b** were created using BioRender.

Our photometry data showed that LH^LepR^ neural population is activated sequentially at seeking and consummatory behaviours. We thought that two hypotheses could be possible; (1) one homogenous LH^LepR^ neuronal population encodes both seeking and consummatory behaviours, or (2) two distinct LH^LepR^ neuron populations encode seeking or consummatory behaviours, respectively. However, individual neural dynamics is not accurately reflected in the fibre photometry. To prove this hypothesis, we investigated changes in LH^LepR^ neural activity using micro-endoscope during seeking and consummatory behaviours (Fig. 3a, Supplementary Movie 5). To distinguish between seeking and consummatory behaviours, we modified the multi-phase test described above (Fig. 2h). During food sessions, fasted mice sequentially performed seeking and consummatory behaviours (Fig. 3b left). In contrast, during no-food sessions, mice performed seeking, but not consummatory behaviours since food was not present in food zone (Fig. 3b right). We identified two distinct neural populations that specifically responded to seeking or consummatory behaviours (Fig. 3c), which was robustly consistent across numerous trials (Supplementary Fig. 4). One population of neurons were activated only during seeking and not during consummatory behaviours (seeking LH^LepR^ neurons) (Fig. 3f–i, Supplementary Fig. 4a). Another population of neurons were activated only during consummatory and not during seeking behaviours (consummatory LH^LepR^ neurons) (Fig. 3j–m, Supplementary Fig. 4b). The two populations were distinctively separated in a 3D scored plot which consists of phase-specific scores that describe the neuronal characteristics (Fig. 3d). Among the population of LH^LepR^ neurons, 25% were seeking neurons, and 39% were consummatory neurons (Fig. 3e). Collectively, our micro-endoscope data showed that seeking LH^LepR^ neurons and consummatory LH^LepR^ neurons; (1), respectively, encode seeking or consummatory behaviours (2) are sequentially activated (Fig. 3c) and are exclusively activated (not simultaneously activated).

### LH^LepR^ neurons fail to evoke eating behaviours in experiments with combination of seeking and consummatory phases

Optogenetic stimulation induces simultaneous activation of both seeking and consummatory LH^LepR^ neurons, which are unphysiological in contrast to our physiological micro-endoscope results. These results imply that optogenetic activation of both seeking and consummatory LH^LepR^ neurons will not induce effective behavioural changes if the mice have choice of both seeking and consummatory behaviours due to competition between two distinct behavioural choices.

**Fig. 4:**
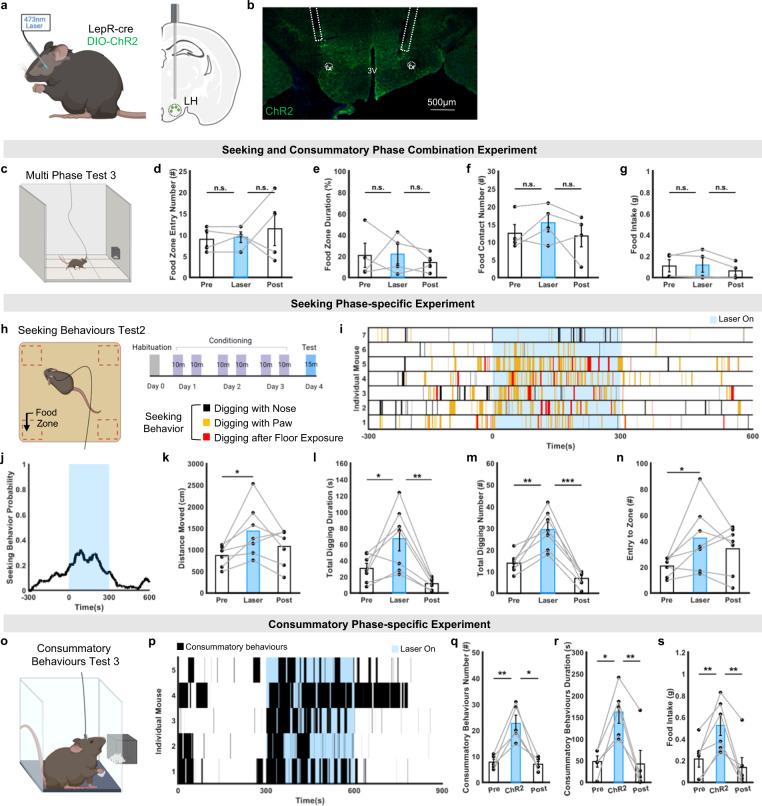
Activation of LH^LepR^ neurons drives seeking or consummatory behaviours. **a**, **b** Schematic of optogenetic activation and image of ChR2 expression in LH^LepR^ neurons. The experiment was repeated at least 4 times independently with similar results. fx, fornix; 3 V, the 3rd ventricle. **c** Schematic of the multi-phase test 3. **d**–**g** Number of food zone entries (**d**), duration in the food zone (**e**), number of food contacts (**f**) and food intakes (**g**) (*n* = 4 mice). Two-sided paired t-test; n.s., *p* > 0.05. **h**, Schematic and schedule of the seeking behaviour test 2. **i** Raster plot during (**h**). **j** Behavioural probability from (**i**). **k**–**n** Quantification of distance moved (**k**), total digging duration (**l**), number of digging behaviours (**m**) and frequency of food zone entries (**n**) (*n* = 7 mice). Two-sided paired t-test; *p = 0.02 (**k**, pre vs laser), *p = 0.04 (**l**, pre vs laser), **p = 0.003 (**l**, laser vs post), **p = 0.002 (**m**, pre vs laser), ***p = 0.00018 (**m**, laser vs post), *p = 0.04 (**n**, pre vs laser). **o** Schematic of the consummatory behaviour test 3. **p**, Raster plot during (**o**). **q**–**s** Number (**q**) and duration (**r**) of consummatory behaviours, and food intake (**s**) (*n* = 5 mice). Two-sided paired t-test; **p = 0.007 (**q**, pre vs laser), *p = 0.0105 (**q**, laser vs post), *p = 0.0105 (**r**, pre vs laser), **p = 0.005 (**r**, laser vs post), *p = 0.015 (**s**, pre vs laser), *p = 0.017 (**s**, laser vs post). Data are represented as mean ± s.e.m. See Supplementary Table 1 for statistics. Source data are provided as a Source data file. The schematics in **a**, **c**, **h** and **o** were created using BioRender.

To examine this hypothesis, LepR cre-mice were injected with cre-dependent channelrhodopsin 2 (ChR2)/halorhodopsin (NpHR) or enhanced yellow fluorescent protein (EYFP) AAV vector, and an optic fibre was implanted in the LH (Fig. 4a, b). We conducted a multi-phase test, in which ad-libitum mice had choice of both seeking and consummatory behaviours in a large chamber (33 × 33 × 33 cm) (Fig. 4c). As expected, unphysiological simultaneous activation/inhibition of both seeking and consummatory LH^LepR^ neurons failed to show any change in seeking (food zone duration and food zone entry number) or consummatory (food contact number and food intake) behaviours (Fig. 4d–g, Supplementary Fig. 5a–j).

### LH^LepR^ neurons evoke seeking or consummatory behaviours in phasic-specific conditions

Our micro-endoscope data distinguished two distinct subpopulation (seeking and consummatory LH^LepR^ neurons) that drive respective behaviours, which are sequentially activated and not simultaneously activated. Therefore, we hypothesised that activation of LH^LepR^ neurons could evoke respective seeking or consummatory behaviours, when the seeking or consummatory phase was isolated so that mice only had a choice of one specific behaviour.

To isolate the seeking phase, mice were conditioned to seek hidden foods in the four corners of an open-field chamber filled with bedding (Fig. 4h, Supplementary Movie 3). On the photostimulation day, ad libitum mice were placed in the same chamber covered with bedding without food to only evoke sustained seeking behaviour. Activation of LH^LepR^ neurons significantly increased seeking behaviours (digging with the nose, digging with the paw, and digging after floor exposure), entry into the food zones, and seeking locomotion compared to no-stimulation (Fig. 4i–n) or control conditions (Supplementary Fig 5k–n). However, inhibition of LH^LepR^ neurons failed to show significant differences in seeking behaviours (Supplementary Fig. 5o–r). Collectively, these results show that LH^LepR^ neurons are sufficient to drive seeking behaviours when the seeking phase is isolated.

To isolate the consummatory phase, ad-libitum mice were placed in a chamber of minimised size (17 × 6 × 30 cm) and were provided with ad libitum food at proximate range (Fig. 4o). Of note, activation of LH^LepR^ neurons significantly increased the number and total duration of consummatory behaviours and food intake when compared to no stimulation (Fig. 4p–s, Supplementary Movie 3). EYFP control mice did not show any significant change in consummatory behaviour (Supplementary Fig. 5s–w). We further performed two validation tests using different behaviour analysis methods to accurately analyse consummatory behaviours. First, by using a manual behaviour analysis method, we revealed that closed-loop stimulation of LH^LepR^ neurons when mice were proximate to food significantly increased consummatory behaviours compared to no stimulation conditions (Supplementary Fig. 6a–d, Supplementary Movie 3). Second, we used a deep-learning-based animal pose estimation method (DeepLabCut)^29^ to computationally extract consummatory behaviours from trials (Supplementary Fig. 6e–i, Supplementary Movie 4). This computerized analysis also validated that stimulating LH^LepR^ neurons significantly evoked consummatory behaviour.

**Fig. 5:**
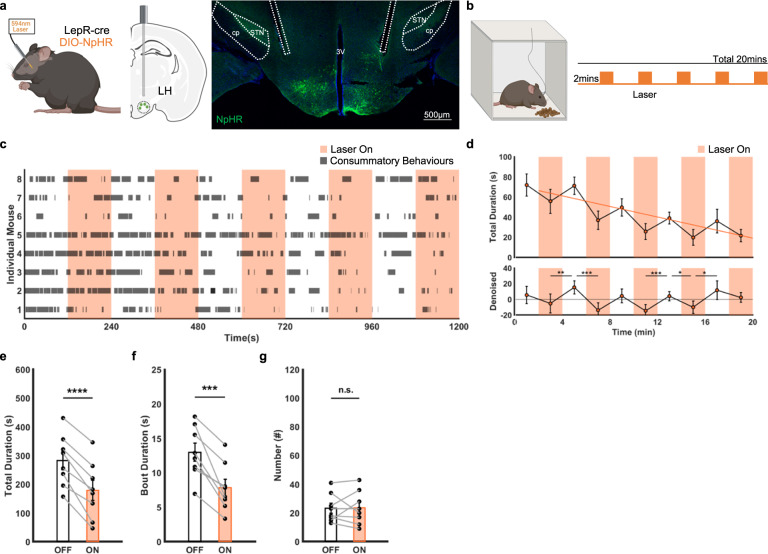
Inhibition of LH^LepR^ neurons decreases consummatory behaviours. **a** Schematic of optogenetic inhibition (left, middle) and image of NpHR expression in LH^LepR^ neurons (right). The experiment was repeated 8 times independently with similar results. 3V, the 3rd ventricle; STN, subthalamic nucleus; cp, cerebral peduncle. **b** Schematic of the consummatory behaviour test 5 and schedule of laser stimulation. **c** Raster plot of consummatory behaviours during (**b**) (*n* = 8 mice). **d** Average duration of consummatory behaviours (top). Calibrated graph (bottom) of the top. Two-sided paired t-test; **p = 0.006 (time bin 2–4 min vs 4–6 min), ***p = 0.0007 (time bin 4–6 min vs 6–8 min), ***p = 0.0009 (time bin 10–12 min vs 12–14 min), *p = 0.02 (time bin 12−14 min vs 14–16 min), *p = 0.019 (time bin 14–16 min vs 16–18 min). **e**–**g** Total duration (**e**), bout duration (**f**), and number (**g**) of consummatory behaviours. Two-sided paired t-test; ****p  < 0.0001 (**e**), ***p = 0.0009 (**f**), n.s., *p* = 0.93 (**g**). Data are mean ± s.e.m. See Supplementary Table 1 for statistics. Source data are provided as a Source data file. The schematics in **a** left and **b** were created using BioRender.

Next, we hypothesised that inhibition of LH^LepR^ neurons decreases consummatory behaviours. Fasted mice were tested in a small chamber where the mice could perform only consummatory behaviours rather than seeking (Figs. 5a, 5b left). To quantify the consummatory behaviour, multiple small snacks were presented during several interleaved photoinhibition blocks (Fig. 5b right, Supplementary Movie 3). During the session, the mice exhibited consummatory behaviours (sniffing, biting and chewing). NpHR mice, but not EYFP control mice, significantly decreased consummatory behaviours (total duration and bout duration) (Fig. 5c-g, Supplementary Fig.7). Collectively, these results show that LH^LepR^ neurons are sufficient and necessary for driving consummatory behaviours when the consummatory phase is isolated.

### NPY increases LH^LepR^ neuron activity via disinhibition of GABAergic interneuron in the LH

**Fig. 6:**
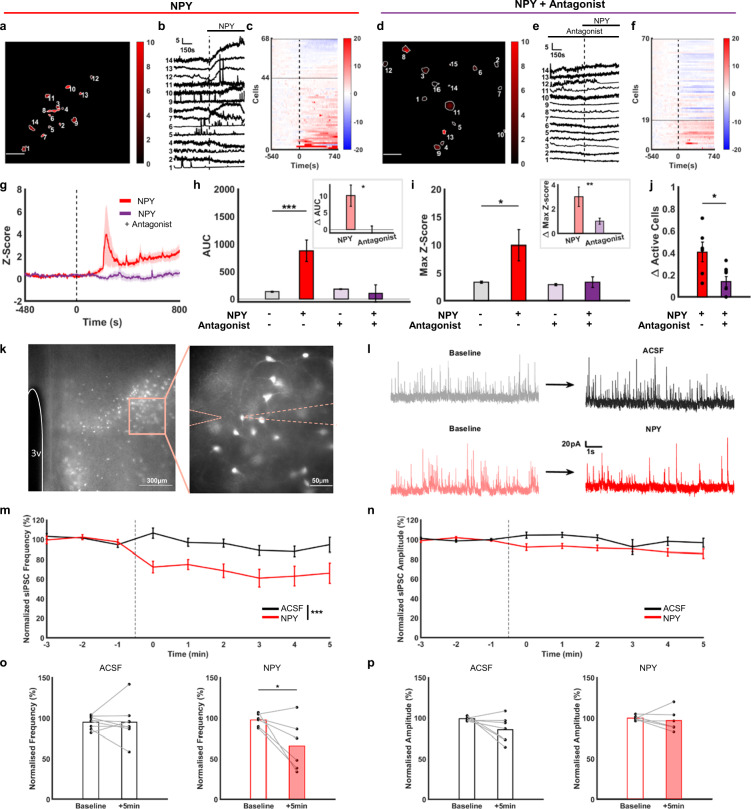
NPY increases LepR neuron activity via disinhibition of GABAergic interneuron in the LH. **a**, **d** Representative image of GCaMP6s-expressing LH^LepR^ neurons during the application of NPY (**a**) or NPY + Antagonist (**d**) using brain slice calcium imaging. The degree of colour brightness represents the degree of cell activity (max Z-score). Scale bar: 50 μm. The experiment was repeated 7 times (**a**) or 9 times (**d**) independently with similar results. **b**, **e** Representative traces of calcium activity of LH^LepR^ neurons marked in (**a** or **d**). Calcium activity aligned to application of NPY (**b**) or NPY + Antagonist (**e**). Dotted black line is start of NPY application. Application scheme is shown on top. **c**, **f** Heatmap depicting calcium signals aligned to application of NPY (**c**) or NPY + Antagonist (**f**). **g** Average Z-score from the LH^LepR^ calcium signal aligned to application of NPY (red) and NPY + Antagonist (purple). **h**, **i** Quantification of AUC (**h**) and max Z-score (**i**) before and after application of NPY or NPY + Antagonist. 68 cells from 7 slices (**a**), 70 cells from 9 slices (**d**). Two-sided paired t-test; ***p = 0.0002 (**h**, left), *p = 0.019 (**i**, left), two-sided unpaired t-test; *p = 0.014 (**h**, right), **p = 0.002 (**i**, right). **j** Quantification of the percentage of active cells. Two-sided unpaired t-test; *p = 0.014. **k** Representative images of td-Tomato-expressing LH^LepR^ neurons during brain slice whole-cell recording. 3 V, the 3rd ventricle. **l** Representative traces of spontaneous inhibitory postsynaptic current comparing ACSF (top) and NPY (bottom). **m**–**p** Time course of sIPSCs frequency (**m**), amplitude (**n**) and quantification of normalised sIPSCs frequency (**o**), amplitude (**p**) in the last 1 min after the ACSF or NPY application. td-Tomato-expressing LH^LepR^ neurons; 8 cells from ACSF, 6 cells from NPY. Two-way repeated measures ANOVA followed by Bonferroni post hoc test (**m**, **n**); ***p = 0.0005 (**m**), two-sided paired t-test (**o**, **p**); *p = 0.043 (**o**, right). Data are mean ± s.e.m. See Supplementary Table 1 for statistics. Source data are provided as a Source data file.

We hypothesised that NPY drives LH^LepR^ neuron activity since (1) a previous study clearly demonstrated that NPY is a key neurotransmitter that drives sustained eating behaviours after the deactivation of AgRP neurons^30^, (2) AgRP/NPY neurons innervate LH^31,32^, (3) NPY receptors are distributed in the LH^33–35^ and (4) the administration of NPY in the LH drives eating behaviours^36,37^. To examine if LH^LepR^ neurons respond to NPY, we performed slice calcium imaging where artificial cerebrospinal fluid (ACSF) with NPY was applied to brain slices containing GCaMP6s-expressing LH^LepR^ neurons (Fig. 6a). As a result, calcium in LH^LepR^ increased after NPY treatment (Fig. 6b, c). The effect of NPY was not observed in the presence of NPY receptor (NPYR) antagonists (Fig. 6d–f). The effect of NPY in the presence of NPYR antagonists was significantly lower than that of NPY alone (Fig. 6g–j). In addition, the effect of NPY on LH^LepR^ neurons was significantly higher than leptin (Supplementary Fig. 8a–d). The response to leptin was heterogenous, which was similar to previous studies^22,24^, reporting that substantial proportion of LH^LepR^ neurons showed decreased activity in response to leptin. Therefore, we speculate that leptin may suppress eating via inhibiting LH^LepR^ neurons.

Since NPY is reported to have an inhibitory effect on neural activity^38,39^, we sought to determine the mechanism underlying the excitatory effect of NPY on LH^LepR^ neurons. According to our analysis of previous single-cell RNA sequencing data^26^, major portion of NPY receptor expressing LH neurons (LH^NPYR^ neurons) were part of a GABAergic population distinct from that of LH^LepR^ neurons, while minor portion co-expressed NPY receptor and leptin receptor neurons (LH^NPYR/LepR^ neurons) (Supplementary Fig. 1l). Therefore, we assumed that the excitatory effects of NPY in LH^LepR^ neurons arose from the major part of the two portions, by disinhibition of presynaptic GABAergic neurons. To confirm whether the activation of LH^LepR^ neurons resulted from a decrease in presynaptic inhibitory inputs, we recorded spontaneous inhibitory postsynaptic currents (sIPSCs) in LH^LepR^ neurons before and after the application of NPY (Fig. 6k, l). The frequency of sIPSCs was significantly decreased with application of NPY, but not ACSF alone (Fig. 6m, o). However, there were no changes in the amplitude of sIPSCs (Fig. 6n, p), suggesting an effect of NPY on presynaptic GABAergic neurons. Overall, these results suggest that NPY increases the activity of LH^LepR^ neurons by decreasing inhibitory inputs.

## Discussion

The present study demonstrated that two distinct LH^LepR^ neuronal populations are activated sequentially and exclusively during the seeking and consummatory eating phases. Further, activation of LH^LepR^ neurons evoked seeking or consummatory behaviours. Neurotransmitter NPY disinhibited LH^LepR^ neurons as a permissive gate signal. Collectively, the present study findings suggest that two distinct LH^LepR^ neuronal populations drive seeking and consummatory behaviours gated by the NPY signal.

Using in vivo micro-endoscope imaging, the present study clearly demonstrated that LH^LepR^ neurons comprise (1) two distinct populations (seeking and consummatory LH^LepR^ neurons), which are sequentially activated during seeking and consummatory behaviours and (2) encode the voluntary drive for eating behaviours. The present study discovered findings compared to previous literature, as follows. A previous micro-endoscope study concluded that LH^LepR^ neurons are one specific population that discriminates between reward cues and non-reward cues^10^. However, this paper did not provide a conclusion regarding different subpopulations among LH^LepR^ neurons. Another previous micro-endoscope study on LH^LepR^ neurons did not classify subpopulation heterogeneity of LH^LepR^ neurons and did not differentiate the different phases of eating^23^. In contrast, applying our comprehensive eating behavioural paradigm, we could successfully distinguish the two distinct populations. In the present study, one population of LH^LepR^ neurons (seeking LH^LepR^ neurons) were only activated during seeking behaviours, not with those for consummatory behaviours. Further, the LH^LepR^ neural activity onset precedes the voluntary seeking behaviour initiation onset, which suggests that LH^LepR^ neurons are drivers of seeking behaviour rather than the consequence of seeking behaviour. The other population of LH^LepR^ neurons (consummatory LH^LepR^ neurons) is only activated during consummatory behaviours, not during seeking behaviours. This population robustly starts to be activated when animals are proximate to food and sustain its activity during consummatory behaviours. These two distinct populations are sequentially activated within the two distinct behavioural phases in eating. For survival, it is crucial for eating behaviours to be correctly sequenced and successfully executed through two distinct phases: seeking (appetitive) and consummatory phases^4,5^. This is equivalent to other motivated behaviours such as social or mating behaviours^6,8^.

Our optogenetics results clearly demonstrated that the causal role of LH^LepR^ neurons in driving seeking and consummatory behaviours via eating phase-specific paradigms. Previous studies reported controversial results that activation of LH^LepR^ neurons decrease eating^16^ or fails to drive eating^10,24^. Another study showed that activation of LH^LepR^ -vlPAG neurons drive eating^23^. To investigate the underlying mechanism of these controversial results, we conducted the following experiments with three phase-specific designs. Since our single-cell resolution results of LH^LepR^ neuron using micro-endoscope robustly distinguished two distinct subpopulations (seeking and consummatory behaviours), we hypothesised that optogenetic stimulation of LH^LepR^ neurons should be conducted during each phase-specific design. As expected, in seeking phase-specific experiments (when only seeking behaviours are possible), LH^LepR^ neurons were sufficient to drive seeking behaviours (searching and digging for expected food). This is consistent with previous results showing that activation of LH^LepR^ neurons increase operant behaviour (lever presses) for food since the operant conditioning test is a one of the seeking-phase-specific experiments^21^. Regarding the consummatory phase-specific experiments (when only consummatory behaviours are possible), as expected, LH^LepR^ neurons are sufficient and necessary for consummatory behaviours only in consummatory phase-specific experiments. On the other hand, in experiments with combination of seeking and consummatory phases (when both seeking and consummatory behaviours are possible), activation of LH^LepR^ neurons failed to evoke seeking or consummatory behaviours (Fig. 4c–g), similar to the previous studies^10^. These phase context-specific optogenetics results provide the neural mechanistic explanation why previous research failed to show increase food intake with large chamber (standard rat/mouse housing cage) experiments where both seeking and consummatory behaviours are possible^10^. These phase context-specific optogenetics results are consistent with our micro-endoscope results regarding two distinct phase-specific activation patterns. These results provide wider understanding of how LH^LepR^ neurons regulate seeking and consummatory behaviours.

Collectively, our data clearly showed that LH^LepR^ neurons fulfil the major criteria necessary to identify them as eating phase specific neurons^1,40^; they are sufficient to drive seeking/consummatory behaviour, they are necessary for consummatory behaviours, LH^LepR^ neurons are activated during seeking and consummatory behaviours. We suggest that the two distinct types of seeking and consummatory LH^LepR^ neurons could have different molecular or connectivity identities. Since voluntary seeking and consummatory behaviours must precede decision-making through the integration of sensory modality information, the medial prefrontal cortex (mPFC) or insular cortex might mediate this process by communicating with LH^LepR^ neurons^41,42^. Seeking and consummatory LH^LepR^ neurons should have distinct upstream and downstream neurons to specifically drive seeking or consummatory behaviours, respectively. Since LH^GABA^-Ventral Tegmental Area (VTA)^14,43^, LH^GABA^-vlPAG^23,44^ and LH^GABA^-Locus Coeruleus (LC)^43^ have been known to mediate eat behaviour, LH^LepR^ seeking or consummatory neurons may innervate VTA, vlPAG or LC. Further, the LH is known to receive input from (mPFC), Orbitofrontal Cortex (OFC), Nucleus Accumbens (NAc), Arcuate Nucleus (ARC) and Nucleus Tractus Solitarii (NTS)^45^. LH^LepR^ neurons also received monosynaptic input from diverse regions such as intra LH, Anterior Cingulate (ACC), Diagonal Band of Broca (DBB), Tuberomammillary Nucleus (TMN) and Ventral Premammillary Nucleus (PMV)^46^. Neural circuit mechanisms related to LH^LepR^ neurons could be elucidated through future studies with activity-dependent tagging, molecular subtyping and projection-specific labelling.

Previously, it was believed that AgRP/NPY neurons directly drive the whole phase of eating behaviours^47,48^. However, recent research has indicated that AgRP/NPY neurons deactivate even in response to a food cue^49^. This suggests that, after the inactivation of AgRP/NPY neurons, another set of neuron drive seeking or consummatory behaviours^50,51^.

In addition, a recent study demonstrated that NPY is a crucial neurotransmitter responsible for sustained hunger after AgRP inactivation^30^. In the present study, the ex vivo results indicated that NPY administration gates LH^LepR^ neurons into an active state via decreasing tonic inhibition (Fig. 6). The effect of NPY was completely abolished in the presence of NPYR antagonist, which further confirms NPYR specific mechanism. This implies that in a sated state, low NPY concentration from low AgRP/NPY neural activity, produces tonic inhibition as a lock, prohibiting LH^LepR^ neurons from responding to food-related cues (Supplementary Fig. 9b). In contrast, in fasted state, high NPY concentration from high AgRP/NPY neural activity unlocks this tonic inhibition and allows LH^LepR^ neurons to generate appropriate eating drive in response to diverse food-related cues. Of note, our in vivo results indicated that LH^LepR^ neuron activity begins to increase in response to seeking (e.g. availability) and consummatory-related cues (e.g. proximate to food), during fasted state (high NPY concentration) (Supplementary Fig. 9b). This internal state-dependent conditional action of eating drive increases rate of survival by restricting eating behaviours only in the fasting state and by avoiding futile and unnecessary behaviours while the animal is sated. Together, our ex vivo results imply that NPY plays a permissive gate role in the manipulation of LH^LepR^ neurons.

Given that a small population of LH^LepR^ neurons co-express NPYR, there may be another alternative mechanism that acts directly through NPY. Both the indirect permissive gate role and the direct role of NPY are not mutually exclusive and could have different complementary roles for the orchestration of LH^LepR^ neuron activity, which requires additional investigation.

The present study provides insight into the role of two distinct LH^LepR^ neurons in orchestrating seeking and consummatory behaviours in eating gated by hunger signal from AgRP/NPY neurons. Understanding the neural circuit mechanism for multi-phase eating behaviours may provide specific treatment options for patients with maladaptive food-seeking and consummatory behaviours.

## Methods

### Animals

All experimental protocols were performed in compliance with the Guide for the Care and Use of Laboratory Animals from the Seoul National University, and approved by the Seoul National University Institutional Animal Care and Use Committee. Mice were housed on a 08:00 to 20:00 light cycle (temperature 22 ± 1 °C, humidity 50 ± 10%) with standard mouse chow (38057, Purina Rodent chow) and water provided ad libitum, unless otherwise noted. Behavioural tests were conducted during the light cycle. Adult male mice (at least 8 weeks old) of the following strains were used: LepR-Cre (JAX stock no.008320), Ai-14 Td-Tomato (JAX stock no. 007914), Vgat-Cre (JAX stock no. 028862)

### sStereotaxic virus injection

Mice were anaesthetised with xylazine (20 mg/kg) and ketamine (120 mg/kg). A pulled-glass pipette was inserted into the LH (400 nl total; AP, −1.5 mm; ML, ±0.9 mm; DV, 5.25 mm from the bregma) based on the 2D LH^LepR^ distribution (Supplementary Fig. 1a–k). The GCaMP6 virus (AAV1.Syn.Flex.GCaMP6s.WPRE.SV40, Addgene 100843; titre: 1.45 × 10^13^ genome copies per ml with 1:2 dilution) was utilised for calcium imaging. The AAV5.EF1α.DIO.hChR2(H134R).EYFP (Addgene 20298; titre: 2.4 × 10^13^ genome copies per ml) or AAV5.EF1α.DIO.eNpHR3.0.EYFP (Addgene 26966; titre: 1.1 × 10^13^ genome copies per ml) or AAV5.EF1α.DIO.EYFP (Addgene 27056; titre: 2.6 × 10^13^ genome copies per ml) was utilised for optogenetic experiments.

### Optical fibre/GRIN lens insertion

For fibre photometry experiments, a ferrule-capped optical cannula (400 µm core, NA 0.57, Doric Lenses, MF2.5, 400/430–0.57) was unilaterally placed 0–50 µm above the virus injection site and attached to the skull with Metabond cement (C&B Super Bond). For optogenetic manipulation, optic fibres (200 µm core, NA 0.37, Doric Lenses or Inper) were bilaterally implanted 100–200 µm above the LH injection site at a 10° angle from the vertical in the lateral-to-medial direction. For micro-endoscope imaging, a GRIN lens (500 µm core, 8.4 length, Inscopix #1050-004413) was inserted after 3 weeks of recovery following virus injection. Dexamethasone, ketoprofen, and cefazolin were administered for postoperative care.

### Calcium imaging using fibre photometry and micro-endoscope

For bulk calcium imaging, we used a Doric Lenses fibre photometry system. In the experiment, 465 nm and 405 nm LED light sources (Doric LED driver) were delivered continuously through a rotary joint (Doric Lenses, FRJ_1×1_PT-400/430/LWMJ-0.57_1m) connected to the patch cord (Doric Lenses, MFP_400/430/1100-0.57_1m), and the GCaMP6 signal was collected back through the same fibre into the photodetector (Doric Lenses). For single-cell calcium imaging, we used nVoke (Inscopix).

### Optogenetics

Laser stimulation (473 nm for activation and 594 nm or 532 nm for inhibition, Shanghai DPSS Laser) was delivered through an FC-FC fibre patch cord (Doric Lenses) connected to the rotary joint, following which the FC-ZF 1.25 fibre patch cord delivered stimulation to the cannula (200 µm core, NA 0.37, Doric Lenses or Inper). The laser intensity was approximately 10 mW at the tip.

### Eating behavioural tests

#### Animal condition

Prior to the experiments, all mice were habituated to the experimental cages, and fibre handling was conducted for at least 3 days. Chocolate-flavoured snack (Oreo O’s, 1/8 aliquot: 0.2 g) was utilised during eating behavioural tests.

#### Multi-phase test 1

The multi-phase test 1 (Fig. 2p) is a behavioural paradigm test with seeking and consummatory phases designed to provide sufficient temporal distinction between seeking and consummatory behaviours. To measure neuronal activity before and after conditioning with food, fasted (80–90% of the body weight in the ad libitum state) mice received a chocolate-flavoured snack at the edge of an L-shaped chamber (60 cm × 8.5 cm) with a shelter (6 cm × 12 cm × 18 cm triangle box). Conditioning sessions (day 1–2) were performed for 15 trials in 2 days to provide sufficient experience for the mice to learn the location of the food by providing a chocolate-flavoured snack. The test session (day 3) was also performed for 15 trials. Each trial started when a door was removed (“accessibility moment”) with scheduled timing from the experimenter. ‘Proximate to food’ was analysed when the mouse arrived at the top of bridge. ‘Food contact’ was defined as the moment when the mouse physically contacted the food. Mice usually entered the shelter spontaneously after each trial (end of consumption). Otherwise, the experimenter closed the door after gently pushing the mice to the shelter.

#### Multi-phase test 2

The multi-phase test 2 (Fig. 1e, f food test first column, Fig. 2h, Fig. 3b) is a behavioural paradigm test which mimicked the natural environment of mice in a cave, running to seek and consume food despite the risk of outdoor threats. To measure the temporal onset of LH^LepR^ neural activity in voluntary behaviour, we eliminated all reward-associated cues (e.g., door open, sound) in the experiment. We placed a shelter as cave and delivered an electrical shock as punishment in a square chamber (30 cm × 30 cm square chamber with narrow corridors sized 6 cm). Electrical shock was given at a mean of 0.2 mA/shock for 7 s with a 10-s interval. We adjusted the total duration of shock delivery to maximise the performance of mice. During conditioning sessions, fasted (80–90% of the body weight in the ad libitum state) mice received a chocolate-flavoured snack at the edge of chamber. During the test session, we exclude the shock and analysed the moment when the mouse’s whole body came out of the shelter (onset of seeking behaviours). Trials that were successful in consuming food were analysed. For micro-endoscope experiments, food and no-food trials were conducted randomly during the test session without shock.

#### Multi-phase test 3

The multi-phase test 3 (Fig. 4c, Supplementary Fig 5a, f) is a behavioural paradigm test which was designed to provide ad libitum accessibility to both seeking and consummatory behaviours, simultaneously. To measure seeking and consummatory behaviours during photostimulation, sucrose agarose gel (30% sucrose in 3% agarose gel) was placed in a food tray (3 cm height) at one side of the open-field box (33 × 33 × 33 cm). Condition of mice was as followed; ad libitum (ChR2)/fasted (NpHR). The food zone was defined as the zone that included the food tray. The size of food zone was defined as approximately 10 cm × 10 cm.

#### Seeking behaviour test 1

The seeking behaviour test 1 (Supplementary Fig. 3a) is a seeking-specific behavioural paradigm test which was designed to evoke only seeking behaviours without any consummatory behaviours. To measure the neural activity during seeking termination, we randomly presented food cue (vertical stripe) and no-food cue (horizontal stripe). During conditioning, fasted (80–90% of the body weight in the ad libitum state) mice received chocolate-flavoured snacks only when the food cue was presented. The success rate ([S2/W], S1 = number of seeking termination, S2 = number of consumptions after food cue, W = S1 + S2) was recorded during training until it reached 80%. The duration and amplitude of shocks during training were optimised for each mouse to achieve the best success rate. During experiment, fasted mice initiated seeking after presentation of the food cue, but eventually terminated voluntarily, when the mice realised there was no food.

#### Seeking behaviour test 2

The seeking behaviour test 2 (Fig. 4h, Supplementary Fig 5k, o) is a seeking specific behavioural paradigm test which was designed to evoke sustained seeking behaviours without any consummatory behaviours. To solely measure seeking behaviours during photostimulation, we conditioned mice to conduct seeking behaviours but removed food at the test day. For the conditioning sessions, chocolate-flavoured snacks or raisins were hidden under the wooden bedding at each edge of the open-field box. Twice a day for 3 consecutive days, the ad libitum mice (ChR2/Control) or fasted mice (NpHR) were allowed to seek the box for hidden food during the 10 mins of the experiment. For the test session, there was only wooden bedding without food in which ad libitum mice (ChR2/Control/NpHR) were put to test. Food zone was defined as four corners divided into 16 zones. Seeking behaviours were analysed in three behaviours manually: digging with nose, digging with paw and digging after floor exposure in food zone.

#### Consummatory behaviour test 1

The consummatory behaviour test 1 is a consummatory specific behavioural paradigm test which was designed to evoke consummatory behaviours with or without swallowing. To measure neural activity during consummatory behaviours, a chocolate-flavoured snack was placed in the tray on one side of the wall. During obtainable height (8 cm) sessions (Fig. 1e, f food test, second column, Fig. 2c), the fasted (80–90% of the body weight in the ad libitum state) mice engaged in sequential consummatory behaviours such as rearing toward visible food, biting, licking, and swallowing. We analysed the moment the mice made physical contact with the hanging food. During the unobtainable height (11 cm) sessions (Supplementary Fig. 3f), the fasted mice initiated consummatory behaviour, rearing toward the visible food, but eventually terminated consummatory behaviours when the mice realised that the mice could not eat it. We analysed the moment the mice voluntarily terminated the consummatory behaviours to the hanging food.

#### Consummatory behaviour test 2

The consummatory behavioural test 2 (Fig. 1e, f food test, third column, non-food test, fourth column; Supplementary Fig. 2f) is a consummatory behavioural paradigm test which was designed to determine whether the neural activity is food specific. To measure neural activity during consummatory behaviour for edible food or inedible non-food objects, fasted (80–90% of the body weight in the ad libitum state) mice performed chewing behaviour toward food (chocolate-flavoured snack) or an inedible object (a Lego brick). We analysed the moment when the mice made physical contact with the food or inedible object.

#### Consummatory behaviour test 3

The consummatory behavioural test 3 (Fig. 4o, Supplementary Fig. 5s–w) is a consummatory specific behavioural paradigm test which was designed to evoke consummatory behaviours without any seeking behaviours. To solely measure consummatory behaviours during photostimulation, we minimised chamber size (17 × 6 × 30 cm). Ad libitum mice (ChR2/Control) or fasted mice (NpHR) were placed in the chamber with sucrose agarose gel (30% sucrose, 3% agarose). During photostimulation, consummatory behaviours were measured; food contact, biting and chewing.

#### Consummatory behaviour test 4

The consummatory behavioural test 4 (Supplementary Fig 6a, e) is a consummatory specific behavioural paradigm test which was designed to evoke consummatory behaviours without any seeking behaviours. To solely measure consummatory behaviours during photostimulation using DeepLabCut behavioural analysis, ad libitum mice were placed in a transparent chamber (10 cm × 10 cm × 15 cm) with a vivid colour food (cheese-flavoured snack). The test was recorded from below (ELP-USB4KHDR01-KV100, no-distortion camera) and from the side (Microsoft LifeCam HD-3000, no-distortion camera). During the testing session, laser stimulation (sham or real) was administered manually for 10 s when the head of the mouse directly faced the food recording the bottom and side views. Bottom and side views of the recorded movies were used for DeepLabCut analysis (24 frames per second). We labelled the snout, mouth (upper jaw, oral commissure, lower jaw), hands, paws, tail base, and food. We trained the network with 960 frames (in the bottom view) or 600 frames (in the side view) using a cut-off of 0.9 p for a total of 500,000 times. For manual behavioural analysis (Supplementary Fig. 6a–d), ad libitum mice were placed in a steel wire cup (10 cm diameter) with a cheese-flavoured snack which is a vivid colour food. The period of consummatory behaviours (licking annotated while tongue was visible, biting) was annotated.

#### Consummatory behaviour test 5

The consummatory behavioural test 5 (Fig. 5b) is a consummatory specific behavioural paradigm test which was designed to evoke discrete short consummatory bouts using small food portions without any seeking behaviours. To solely measure consummatory behaviours during photostimulation, we conducted experiment in a minimised chamber size (13 × 17 × 30 cm). The mice (fasted 16–24 h) were given ad libitum chocolate-flavoured snacks. On the test day, laser stimulation was delivered for 20 min at 2-min intervals.

#### Water test

Mice were dehydrated for 2 days. The water test was performed using an open-field chamber where a water bottle was placed. We analysed the moment when mice licked the spout of the water bottle.

### 3D clearing

Fixed tissue was incubated in reflective index matching solution (C Match, Cat.50-3011) at 37 °C for 2 days. Images were obtained using SPIM (LaVision Biotech, Bielefeld, Germany) and analysed using IMARIS 9.5 (Bitplane AG, Zürich, Switzerland).

### Calcium imaging of brain slices

Brain was extracted after mice were decapitated under isoflurane anaesthesia at least 3 weeks after virus injection. LH slices were dissected to a thickness of 250 μm using a vibratome (VT1200S, Leica, Nussloch, Germany) in ice-cold standard artificial cerebrospinal fluid (ACSF) containing the following (in mM): 125 NaCl, 2.5 KCl, 1 MgCl_2_, 2 CaCl_2_, 1.25 NaH_2_PO_4_, 26 NaHCO_3_, and 10 glucose, bubbled with 95% O_2_ and 5% CO_2_. For recovery, the slices were incubated at 32 °C for 15 min and then further incubated for 1 h at room temperature. The slices were then transferred to the recording chamber and perfused with ACSF at 32 °C during imaging. Calcium measurements were performed using a CMOS camera (Photometrics, Tucson, AZ) attached to an upright microscope (BX50WI, Olympus, Tokyo, Japan) with a 40X or 10X water-immersion objective (NA 0.8 or 0.3, LUMPlanFL N or UMPlanFl; Olympus) at 10 frames per second. A broad white light source (pE-340 Fura, CoolLED, Andover, UK) was passed through an excitation filter (450–480 nm) and collected through an emission filter (525/50 nm). Fluorescence images were acquired using VisiView software (Visitron Systems GmbH, Puchheim Germany).

### Whole-cell patch-clamp recording

Brain was extracted after mice were decapitated under isoflurane anesthesia from LepR-tdTomato mice. LH slices were dissected to a thickness of 250 μm using a vibratome (Leica, VT1200S) with carbogen-saturated (95% O_2_ and 5% CO_2_) sucrose solution containing the following (in mM): 75 NaCl, 75 sucrose, 25 glucose, 26 NaHCO_3_, 7 MgCl_2_, 2.5 KCl, 1.25 NaH_2_PO_4_, and 0.5 CaCl_2_. For recovery, slices were incubated at 32 °C for 15 min in standard ACSF containing the following (in mM): 125 NaCl, 2.5 KCl, 1 MgCl_2_, 2 CaCl_2_, 1.25 NaH_2_PO_4_, 26 NaHCO_3_, and 10 glucose, bubbled with 95% O_2_ and 5% CO_2_. Following further incubation for 1 h at room temperature, the slices were transferred to the recording chamber and perfused with ACSF at 32 °C during recording. Whole-cell patch-clamp recordings were performed in LH neurons expressing tdTomato using EPC9 (HEKA, Ludwigshafen am Rhein, Germany). The resistance of the pipette was 2–5 MΩ when filled with an intracellular solution containing the following (in mM): 135 CsMS, 10 CsCl, 10 HEPES, 0.2 EGTA, 4 Na_2_-ATP, and 0.4 Na_3_-GTP (pH 7.2–7.3). All electrophysiological recordings were started at least 4 mins after the whole-cell configuration had been established.

### Drugs

Neuropeptide Y (NPY), BIBO 3304 trifluoroacetate (NPY Y1 receptor antagonist) and CGP 71683 hydrochloride (NPY Y5 receptor antagonist) were purchased from Tocris (Bristol, UK). Recombinant mouse Leptin Protein was purchased from R&D systems (Minneapolis, MN). These were dissolved in ACSF for slice application. The drug concentrations used in slice Ca2+ imaging experiments were as follows: 1 µM NPY, 1 µM BIBO 3304 trifluoroacetate, 10 µM CGP 71683 hydrochloride and 100 nM Recombinant mouse Leptin Protein. The drug concentration of NPY used in whole-cell recording with pico-pump was 2 µM.

### Histology, immunohistochemistry and imaging

Animals were deeply anesthetized by a mixture of ketamine and xylazine. Transcranial perfusion was performed using phosphate-buffered saline, followed by 4% neutral-buffered paraformaldehyde (T&I, BPP-9004). The brains were extracted, post-fixed in 4% paraformaldehyde at 4 °C, and transferred to 10% sucrose, followed by 30% sucrose for cryoprotection. Cryoprotected brains were sectioned coronally on a cryostat (Leica Biosystems, CM3050) at 50 µm, and their sections were stained with 4’,6-diamidino-2-phenylindole (DAPI) to visualise the nuclei. To verify the scientific exactitude, images of viral fluorescence and fibre/cannula placement were captured using a confocal microscope (Olympus, FV3000).

### Analysis

#### Single-cell RNA-sequence analysis

scRNA-sequence data with the LH (GSE125065) were analysed^26^. Of the initial 7232 cells (3439 male and 3793 female), 598 cells with less than 500 unique molecular identifiers (UMIs) or >40% of mitochondrial reads were discarded. The R package Monocle3 was used to classify the cells^52^. Using Monocle 3, we subjected single-cell gene expression profiles to uniform manifold approximation and projection (UMAP) visualisation. Altogether, we identified 4091 cells as neural clusters on the basis of cell type-specific marker gene expression^26,52,53^. These neural clusters containing 4091 cells were extracted for further clustering using Monocle 3 as above, which yielded 37 clusters. Clusters were classified as GABAergic when the median expression of Slc32a1 was greater than that of Slc17a6 in each cluster and glutamatergic when the median expression of Slc17a6 was greater than that of Slc32a1. Consistent with previous result (92%^21^, 80%^23^), most of LH^LepR^ neurons were GABAergic (70%; 100/141).

#### Simulated distribution of food-specific LH^LepR^ neurons among LH^GABA^ neurons

Simulated results of 1000 LH^GABA^ neurons. We assumed that 10% of LH^GABA^ neurons are LH^LepR^ neurons, given that our result (Supplementary Fig. 1m) and a previous result^21^ indicated that LH^LepR^ neurons constitute 4–20% of LH^GABA^ neurons. In our result, among LH^GABA^ neurons, 8% of LH^GABA^ neurons were food specific (80 neurons) (Fig. 1i). Among LH^LepR^ neurons (10% of 1000 LH^GABA^ neurons, 100 neurons), 63% of LH^LepR^ neurons were food specific (63 neurons) (Fig. 1l). Therefore, LH^LepR^ neurons comprise the majority of food-specific LH^GABA^ neurons (79%; 63/80) in this simulation results.

#### Behavioural tests

All data analyses were performed using custom-written MATLAB (MathWords, Natick, MA) and Python codes. Behavioural experiments were analysed using Observer XT 13 or EthoVision 14 or DeepLabCut.

#### Computational extraction of consummatory behaviours

To distinguish consummatory behaviours from other behaviours, we defined three criteria (Supplementary Movie 4). In the bottom view, mice consuming the cheese-flavoured snacks stood up slightly, and the distance between the front paws decreased because they had been brought together to catch the food. Therefore, the first criterion was met when the distance between the left and right front paws was less than that between the left and right hind paws in the bottom view. The second criterion was met when the y-coordinates decreased sequentially for the tail base, midpoint of the two hind paws, and midpoint of the two front paws in the bottom view. The third criterion was met when the snout coordinates were in the food zone in both the bottom and side views.

#### Quantification of neural onset

The neural onset can be determined by differentiating the neural activity recorded from calcium signals and analysing the maximum value of the third derivative of the neural activity^27,28^. For each trial, neural activity that was computed into the Z-score with a baseline (–10 s to –5 s from seeking), was fitted to the optimal polynomial degree for differentiation. We calculated 1^st^ derivative of neural activity, which is the velocity of neural activity. From the velocity, we calculated the 2nd derivative, which is the acceleration of neuronal activity. From the acceleration, we calculated the 3^rd^ derivative, which is the jolt of neural activity. By calculating the time point when jolt (3^rd^ derivative) reaches its maximum value, we could calculate the mathematical signal onset when the signal begins to increase. The optimal polynomial degree was determined manually according to traces that best fit the computed trace. Then, the third derivative of the fitted neural activity was calculated. Afterwards, the optimal time of the maximum value of the third derivative around the neural onset was determined manually and quantified into the latency to the seeking behavioural onset (Fig. 2m–o).

#### Fibre photometry imaging

Fibre photometry signal data were acquired using the Doric Studio software. Two signals from fibre photometry, 465 nm calcium and 405 nm isosbestic signals (for artefact correction), were obtained for correction before performing any analysis. Signals from fibre photometry were corrected as follows to minimise artefact recordings: corrected 465 nm signal = (465 nm signal − 405 nm signal)/405 nm signal^54^. Signals were decimated to obtain approximately 25 data points in 1 s. For photometry experiments, all corrected signals shown were initially computed to Z-scores before further normalisation. The baseline was designated as –10 s to –5 s before recording the initiation of behaviour (t = 0). The mean of the baseline (m) and standard deviation (σ) of the baseline were computed to normalise the corrected signals into Z-scores (Z = (corrected 465 nm − m)/σ). The behaviour time point for each test was manually annotated. For the heatmap, each trial was normalised before visualisation (normalised Z = (Z − minimum Z)/(max Z − min Z)). Trials were excluded if the trial length exceeded the optimal trial length (15 s for the multi-phase, 10 s for the rest).

#### Micro-endoscopic imaging

All data from the micro-endoscope experiments were recorded using nVoke (Inscopix). The raw signal output from CNMF-E (Craw) was converted into Z-scores (Z = (Craw − m)/σ), according to the mean (m) and standard deviation (σ) of the baseline (−10 s to −5 s before behavioural initiation).

To discriminate food-specific neurons in Fig. 1, we applied the following criteria. We defined neurons as food-specific responsive(yellow) when they were activated during all three eating behavioural tests (>4σ) and not activated during a non-food behavioural test (<4σ). We defined neurons as non-specific-responsive (grey) when they were both activated during three eating behavioural tests (>4σ) and a non-food behavioural test (>4σ). We defined neurons as non-food-specific responsive (blue) when they were not activated during all three eating behavioural tests (<4σ) but, activated during a non-food behavioural test (>4σ). We defined neurons as no responsive (white) when they were neither activated during three eating tests nor a non-food behavioural test (<4σ).

To distinguish the distinct populations of LH^LepR^ neurons in Fig. 3, the neural activity of LH^LepR^ neurons was recorded in multi-phase test 2 and processed as described above. Trials that exceeded 25 s of total trial length were excluded from the test (seeking moment – food consumption end [food trial] or food zone exit [no food trial]). Activated neurons were defined as cells with Z-scores of >4σ. Otherwise, we defined non-responsive neurons if neural activity was Z-scores of <4σ. Neural activity was then normalised as follows: (NF0) = (Craw − minimum Craw)/(max Craw − minimum Craw). Further analysis was performed with the average normalised activity of the group of trials that had sufficient length. Seeking-score-1 was defined as NF0 at the food contact moment in the seeking with consummatory behaviour session. Seeking-score-2 was defined as NF0 at the food contact moment in the seeking without consummatory session. Consummatory score was defined as the difference in the value of NF0 at the last moment of the trial and seeking-score-1. Neurons that had higher seeking-score-1 than consummatory score and a seeking-score-2 higher than NF0 0.4 were defined as seeking neurons. Neurons that had higher consummatory score than seeking-score-1, or if seeking-score-2 was lower than NF0 0.4 were defined as consummatory neurons. Other neurons were defined as ambiguous neurons.

#### Brain slice calcium imaging

For brain slice data, first 2 min of slice data were excluded from analysis to exclude the photobleaching effect of the camera during the first minutes of the recording. Z-score was computed as stated above (baseline: −480 s ~ 0 s from drug delivery), then, linear regression of the mean of baseline of each brain slice activity was computed (mean trace from all cell traces from the brain slice) and subtracted from the cell activity trace to account for the photobleaching or brain slice movement effect during the experiment. Afterwards, cells were defined excitatory if neural activity exceeded the adjusted Z-score of 4σ. Due to the lack of spontaneous activity of LH^LepR^ neurons, decreased neural activity was not analysed in slice Ca^2^+ imaging.

#### Whole-cell patch-clamp recording

Spontaneous inhibitory postsynaptic currents (sIPSCs) were analysed using the Minhee Analysis Package^55^. The effects of NPY on sIPSCs were analysed by measuring the percentage change, compared to baseline for each neuron. The neurons in which the change was higher than 20% from the baseline were discarded, and those that exhibited sIPSCs over 5 Hz were used.

#### Statistical analysis

All statistical data were analysed using MATLAB or IBM SPSS 25.0 (IBM Corp., Armonk, NY). Data in the figures are reported as the mean ± standard error of the mean. Paired t-tests were used to compare data between two groups. Two-way repeated-measures analyses of variance (ANOVA) were used for multiple comparisons. *P*-values for comparisons across multiple groups were corrected using the Greenhouse–Geisser method in IBM SPSS 25.0. Levels of significance were as follows: *p < 0.05. **p < 0.01, ***p < 0.001, ****p < 0.0001.

### Reporting summary

Further information on research design is available in the Nature Portfolio Reporting Summary linked to this article.

## Supplementary information

Supplementary Information

Description of Additional Supplementary Files

Supplementary Movie 1

Supplementary Movie 2

Supplementary Movie 3

Supplementary Movie 4

Supplementary Movie 5

Reporting Summary

## Data Availability

The neural and behavioural data used to generate the figure in this study has been deposited in Figshare repository under accession code 10.6084/m9.figshare.22058123.v2.

## Code availability

Custom Matlab code to generate figures in this study has been deposited in Figshare repository 10.6084/m9.figshare.22058123.v2. Code for patch clamp recording data is available at https://github.com/parkgilbong/Minhee_Analysis_Pack.
