# Supplementary material for: Lateral hypothalamic leptin receptor neurons drive hunger-gated food-seeking and consummatory behaviours in male mice
Source: Nat Commun. 2023 Mar 17;14:1486. doi: 10.1038/s41467-023-37044-4 (PMC10023672; doi:10.1038/s41467-023-37044-4)
Supplement: Supplementary file 1 — Supplementary Information [file 41467_2023_37044_MOESM1_ESM.pdf]

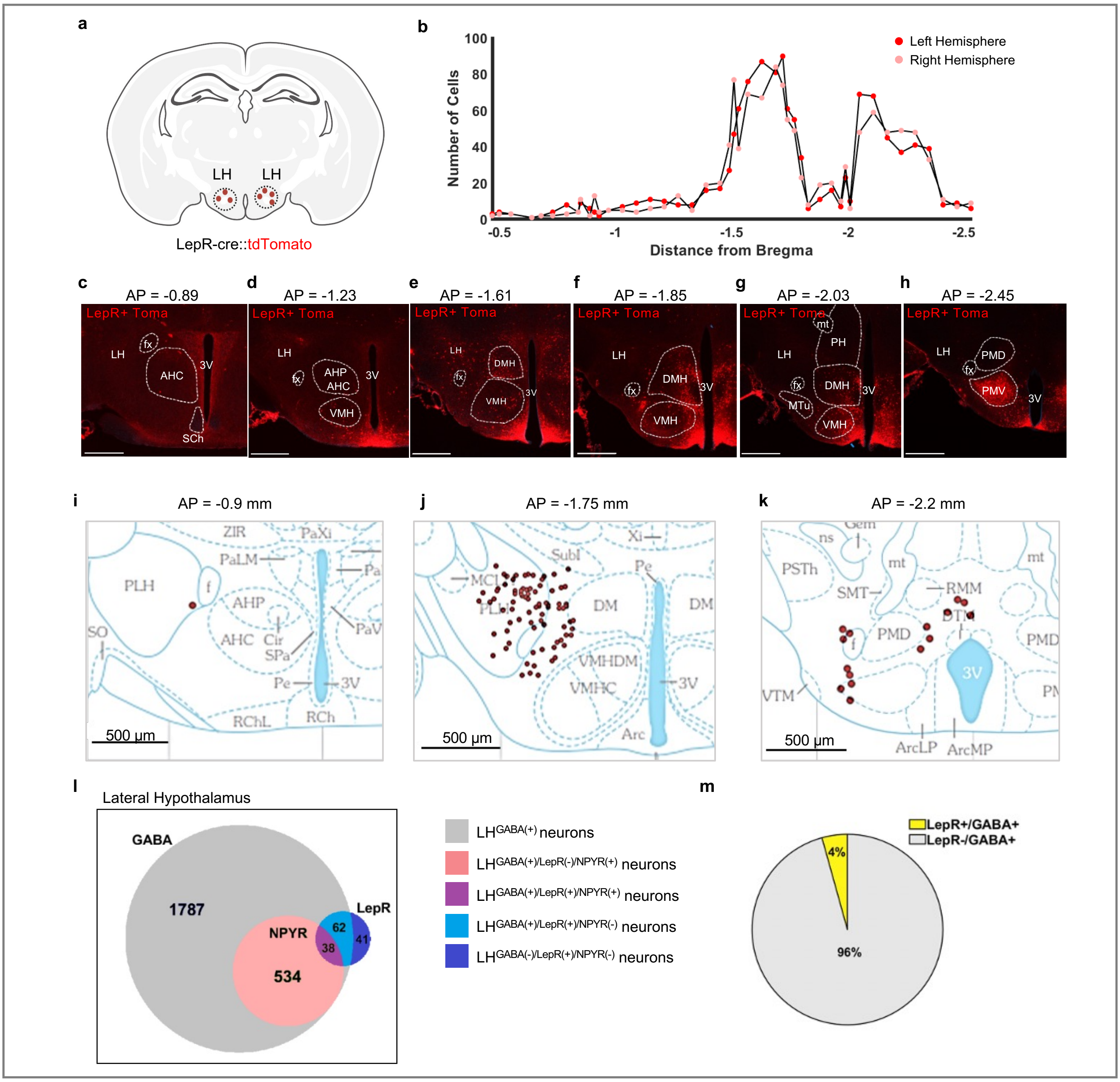

Supplementary Figure. 1 | Distribution and molecular identity of LepR neural population in the lateral hypothalamus.
a, b, Distribution profile and average quantification of LepR-positive cells in the LH along the anterior-posterior axis of LepR-tdTomato mice (n = 3 mice). **c-h**, Coronal brain sections showing tdTomato+ cells. Scale bar: 500µm. The experiment was repeated 3 times independently with similar results. fx, fornix; 3V, the 3rd ventricle; AHC, anterior hypothalamus central; SCh, suprachiasmatic nucleus; AHP, anterior hypothalamus posterior; VMH, ventromedial hypothalamus; DMH, dorsomedial hypothalamus; PH, posterior hypothalamus; MTu, medial tuberal nucleus; mt, mamillothalamic tract; PMD, premamillary nucleus dorsal part; PMV, premamillary nucleus ventral part. **i-k**, Representative images depicting the distribution of LH^{LepR} tdTomato+ cell bodies (red dot). **l**, Venn diagram of molecular characteristics (GABA, NPYR, LepR) of the LH neurons based on single-cell RNA sequencing data. **m**, Proportion of LepR-positive (yellow) and LepR-negative (grey) neurons among GABA-positive neurons based on single-cell RNA sequencing data. Source data are provided as a Source Data file. .The schematics in a were created using BioRender.

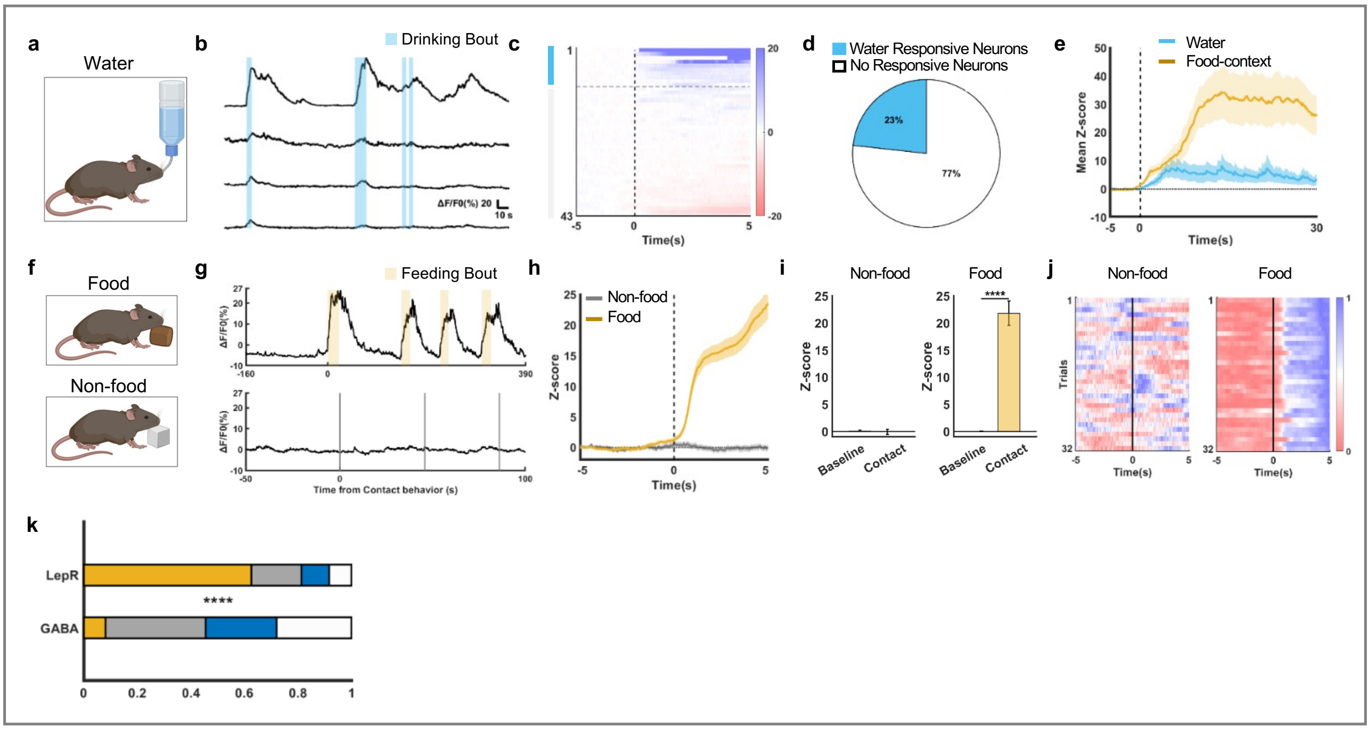

Supplementary Figure. 2 | Activity of LH^{LepR} neurons is food-specific.
a, Schematic of the behavioural test for drinking water in the dehydration state. **b**, Representative single-cell traces of LH^{LepR} neurons. The blue shaded box indicates each bout of drinking. **c**, Heatmap depicting calcium signals aligned with drinking behaviour. Water-responsive cells (blue) activated (>4σ) during drinking water. (LH^{LepR} neurons 48 cells, 4 mice). **d**, Proportion of water-responsive cells (blue, 23%) and no responsive cells (white, 77%). **e**, Average Z-score from the LH^{LepR} calcium signal aligned to drinking behaviour for water (blue) and feeding behaviour for food (yellow). **f**, Schematic of consummatory behaviour test 2 (food/non-food). **g**, Representative calcium traces from the LH^{LepR} calcium signal during (**f**). The yellow shaded box indicates behaviour from food contact to end of consumption, and the grey line indicates contact with non-food. **h**, Average Z-score from the LH^{LepR} calcium signal aligned to contact with food (yellow) and non-food (grey). **i**, Quantification of the Z-score in calcium signal changes from (**h**). Comparison between baseline (-8 to -7s) and after contact (4 to 5s). (4 mice; 32 trials). **j**, Heatmap depicting the normalised LH^{LepR} calcium signal aligned to contact with non-food and food. **k**, Comparison between the proportion of LH^{GABA} neurons and proportion of LH^{LepR} neurons for food-specific neurons responsive (yellow), non-specific responsive (grey), non-food-specific responsive (blue), and non-responsive (white) cells. Chi-square statistic of cross-tabulation table; **** $p < 0.0001$. Data are mean ± s.e.m. See Supplementary Table 1 for statistics. Source data are provided as a Source Data file. The schematics in a, and f were created using BioRender.

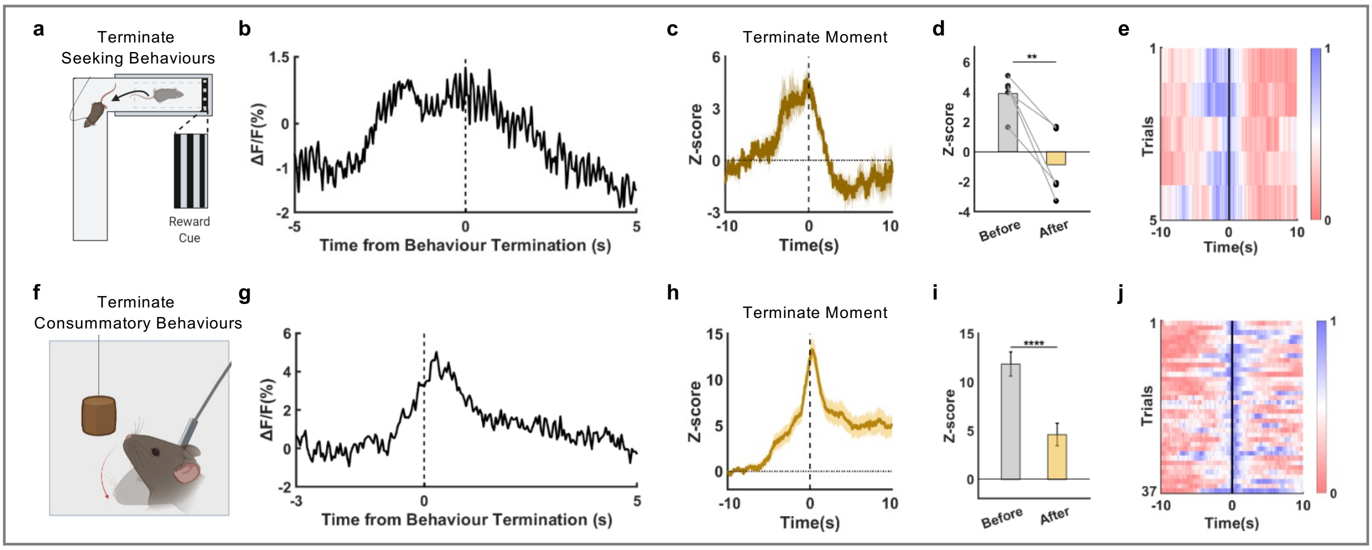

Supplementary Figure. 3 | LH^{LepR} neurons are inactivated at termination moment of seeking and consummatory behaviours.
a, **f**, Schematic of the seeking behaviour test 1 (**a**) and consummatory behaviour test 1 (unobtainable) (**f**). **b**, **g**, Representative calcium signal of LH^{LepR} neurons aligned to the termination moment of seeking (**b**) and consummatory (**g**) behaviours. **c**, **h**, Average Z-score from LH^{LepR} neurons aligned to the termination moment of seeking (**c**) and consummatory (**h**) behaviours. **d**, **i**, Quantification of the Z-score from (**c**, **h**). Comparison between the before (0 to 1s) and (9–10s) after behavioural termination. (**d:** 1 mouse, 5 trials) (**i:** 5 mice, 37 trials). **e**, **j**, Heatmap depicting the normalised LH^{LepR} calcium signal aligned to the termination moment of seeking (**e**) and consummatory (**j**) behaviours. Data are mean ± s.e.m. See Supplementary Table 1 for statistics. Source data are provided as a Source Data file. The schematics in a and f were created using BioRender.

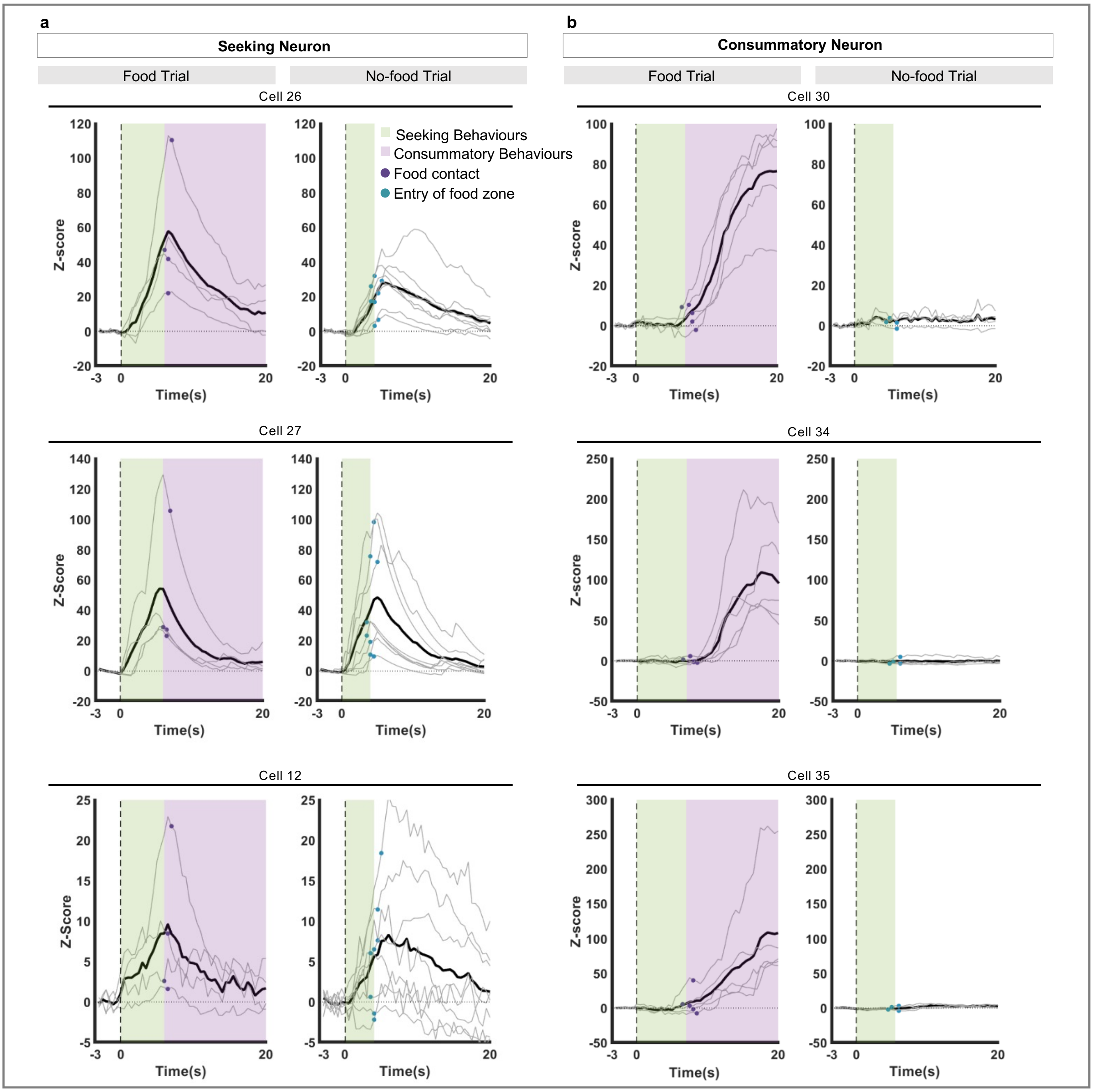

Supplementary Figure. 4 | The activities of seeking and consummatory LH^{LepR} neurons are time-locked to seeking and consummatory behaviours, respectively with robust consistency over multiple trials.

a, **b**, Representative activity of an individual seeking neurons (**a**) and consummatory neurons (**b**) during multi-phase test 2. Average Z-score (bold) aligned to the seeking initiation moment. The grey lines indicate the calcium signal of individual trials. The violet dot represents the moment of physical food contact (initiation of consummatory behaviours). The blue dot represents the moment of entry into the food zone. The green shade denotes seeking behaviours and the purple shade denotes consummatory behaviours. Source data are provided as a Source Data file.

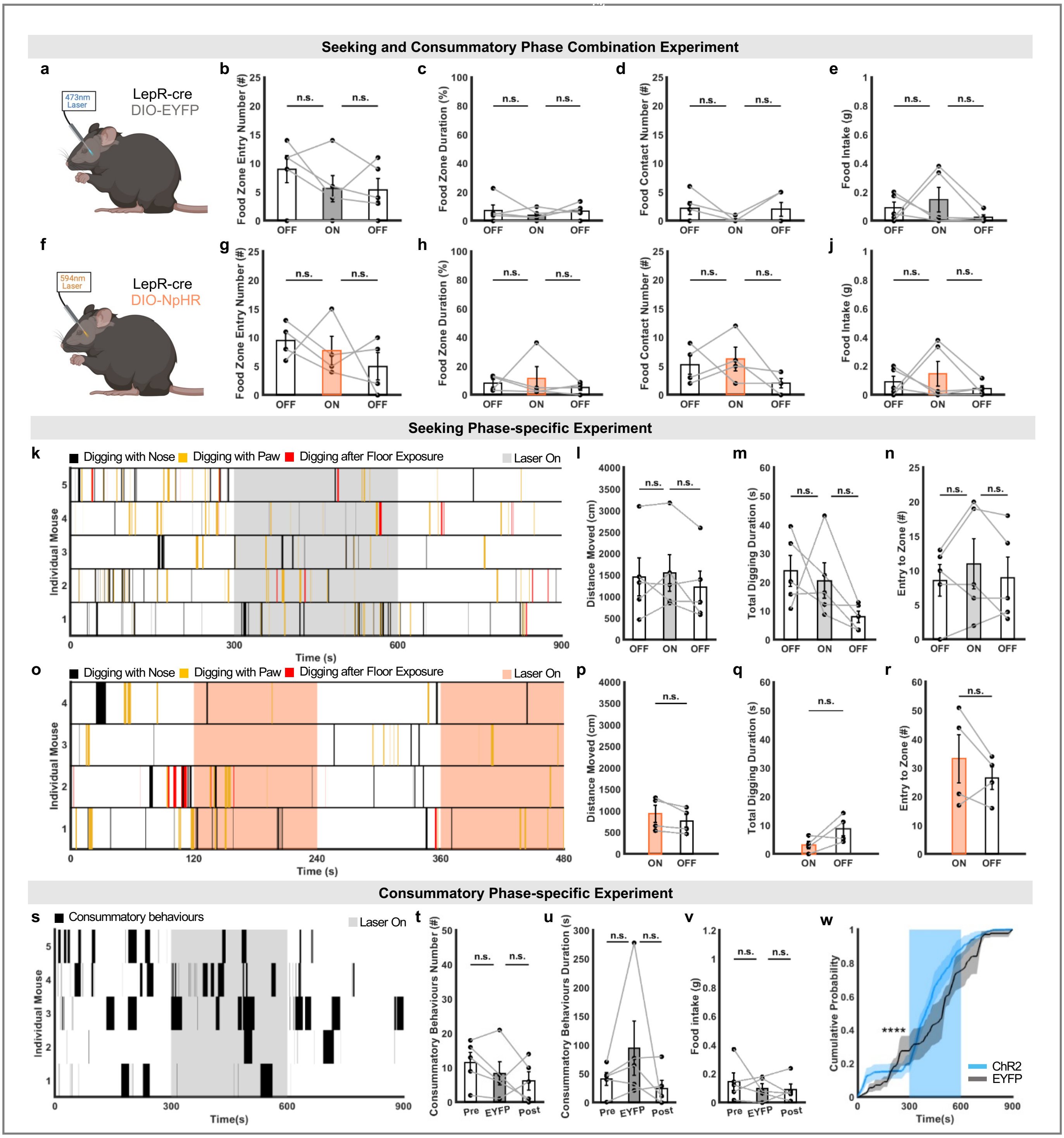

Supplementary Figure. 5 | Control Experiments for Figure 4.

a, f, Schematic of optogenetic control (**a**) and inhibition (**f**) of LH^{LepR} neurons during multi-phase test 3. **b-e**, Number of food zone entries (**b**), duration in the food zone (**c**), number of food contacts (**d**) and food intakes (**e**) from EYFP-injected mice (n = 4 mice). Two-sided paired t-test; n.s., p > 0.05 . **g-i**, Number of food zone entries (**g**), duration in the food zone (**h**), number of food contacts (**i**) and food intakes (**j**) from NpHR-injected mice (n = 4 mice). Two-sided paired t-test; n.s., p > 0.05. **k, o**, Raster plot from EYFP-injected mice (**k**) and NpHR-injected mice (**o**) during the seeking behaviour test 2. (n = 5 mice ; n = 4 mice). **l-n**, Quantification of distance moved (**l**), total digging duration (**m**), and frequency of food zone entries (**n**) from EYFP-injected mice. Two-sided paired t-test; n.s., p > 0.05. **p-r**, Quantification of distance moved (**p**), total digging duration (**q**), and frequency of food zone entries (**r**) from NpHR-injected mice. Two-sided paired t-test; n.s., p > 0.05. **s**, Raster plot from EYFP-injected mice during the consummatory behaviour test 3 (n = 5 mice). **t-v**, Number (**t**) and duration (**u**) of consummatory behaviours, and food intake (**v**). Two-sided paired t-test; n.s., p > 0.05. **w**, Cumulative probability of consummatory behaviours between ChR2-injected mice and EYFP-injected mice. Two sample Kolmogorov-smirnov test; **** p < 0.0001. Data mean ± s.e.m. See Supplementary Table 1 for statistics. Source data are provided as a Source Data file. The schematics in a and f were created using BioRender.

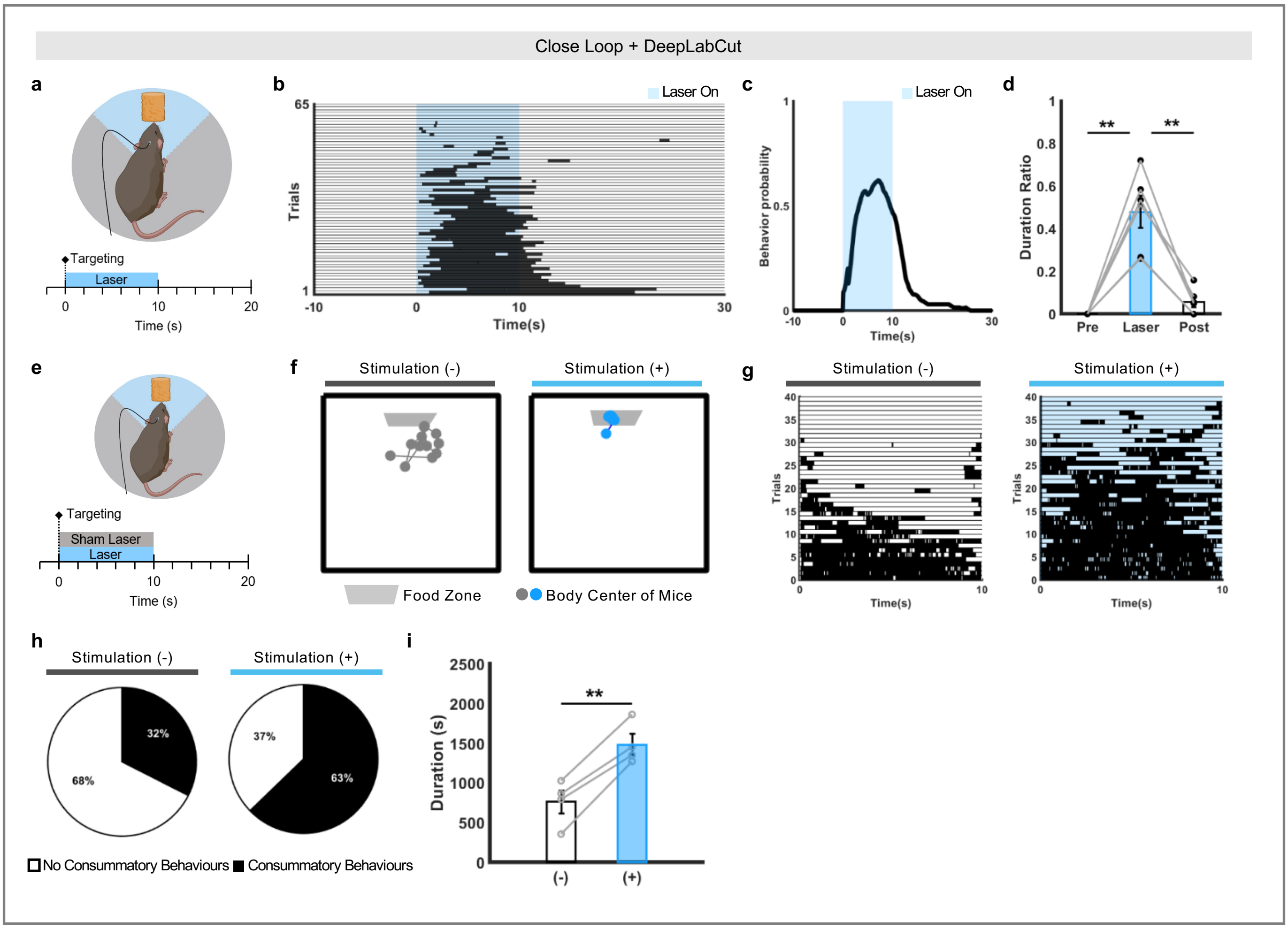

Supplementary Figure. 6 | Activation of LH^LepR neurons drives consummatory behaviours.
a, Schematic of the consummatory behaviour test 4. The laser is stimulated when the head of the mouse is in the food zone (blue). **b**, **c**, Raster plot (**b**) and behavioural probability (**c**) of consummatory behaviours (n = 6 mice, 65 trials). **d**, Quantification of consummatory behaviours from (**c**). Two-sided paired t-test; ** p = 0.0014 (pre vs laser), ** p = 0.0013 (laser vs post). **e**, Schematic of laser or sham laser stimulation during the consummatory behaviour test 4. **f**, Trajectory of the centroid of the snout, left hand, and right hand in bottom view using DeepLabCut. **g**, Raster plot during (**e**). Black squares indicate consummatory behaviours from the DeepLabCut behaviours analysis. **h**, Proportion of consummatory behaviours from (**g**) (4 mice). **i**, Quantification of consummatory behaviours from (**g**) (n = 4 mice) Two-sided paired t-test; ** p = 0.0038. Data are mean ± s.e.m. See Supplementary Table 1 for statistics. Source data are provided as a Source Data file. The schematics in a and e were created using BioRender.

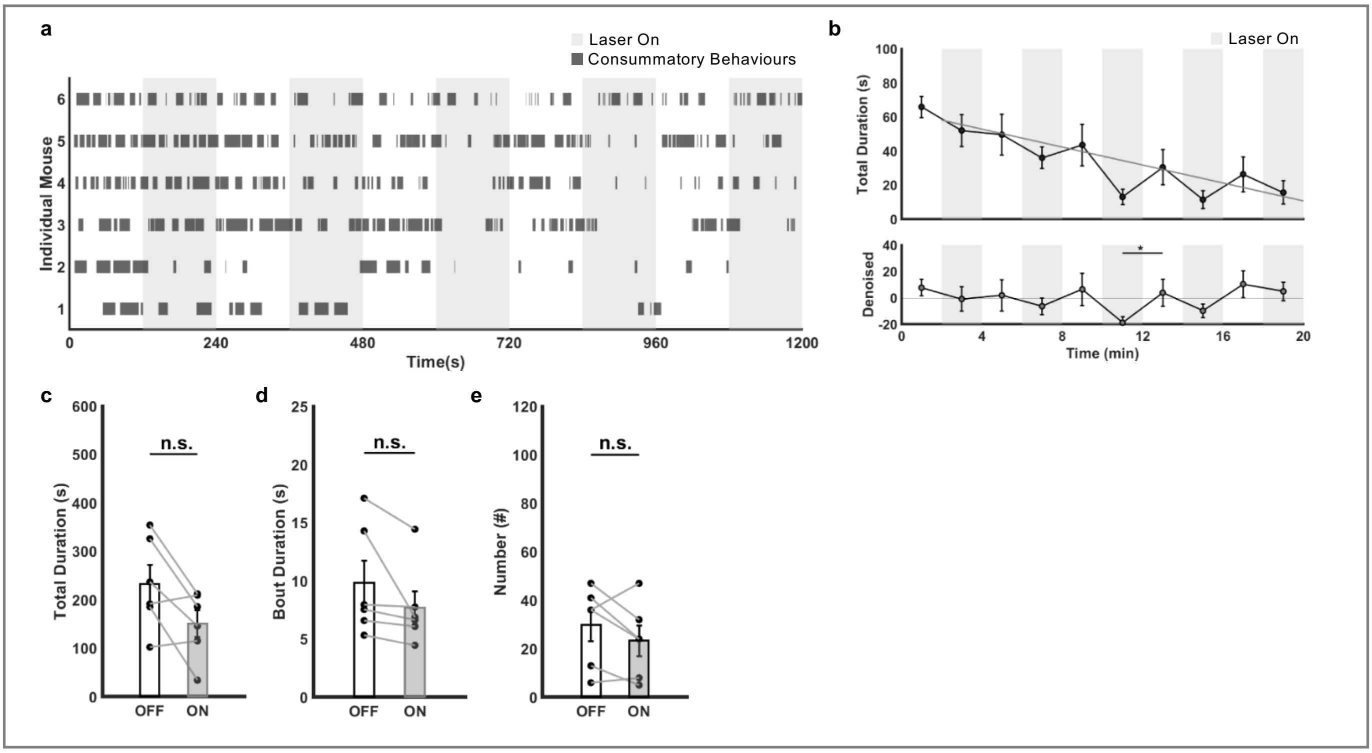

Supplementary Figure. 7 | Control experiments for figure 5.

a, Raster plot of consummatory behaviours from EYFP-injected mice during consummatory behaviour test 5 (n = 6 mice). **b**, Average duration of consummatory behaviours (top). Calibrated graph (bottom) of top. **c-e**, Quantification from (**a**). Total duration (**c**), bout duration (**d**), number (**e**) of consummatory behaviours. Two-sided paired t-test (**b-e**); * p = 0.04 (**b**, time bin 10-12 min vs 12-14 min), n.s., p = 0.0502 (**c**), p = 0.12 (**d**), p = 0.2 (**e**). Data are mean ± s.e.m. See Supplementary Table 1 for statistics. Source data are provided as a Source Data file.

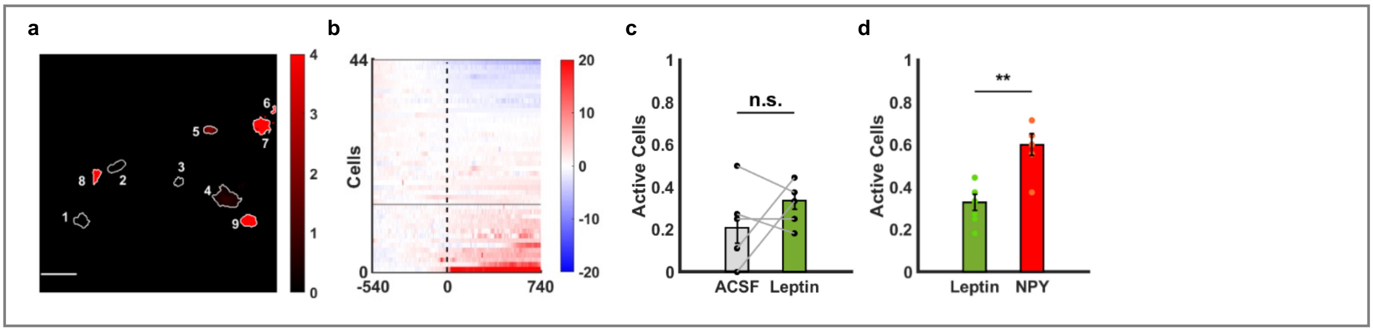

Supplementary Figure. 8 | LepR neurons respond heterogeneously to leptin in the LH.
a, Representative image of GCaMP6s-expressing LH^{LepR} neurons during the application of leptin using brain slice calcium imaging. Scale bar: 50µm. The experiment was repeated 6 times independently with similar results. **b**, Heatmap depicting calcium signals aligned to application of leptin. Dotted black line is start of leptin application. 44 cells from 6 slices. **c**, Quantification of the percentage of active cells before (grey) and after (green) application of leptin. Two-sided paired t-test; n.s., p = 0.21. **d**, Quantification of the percentage of active cell during application of leptin (green, 6 slices) and NPY (red, 7 slices). Two-sided unpaired t-test; ** p = 0.001. Data are mean ± s.e.m. See Supplementary Table 1 for statistics.Source data are provided as a Source Data file.

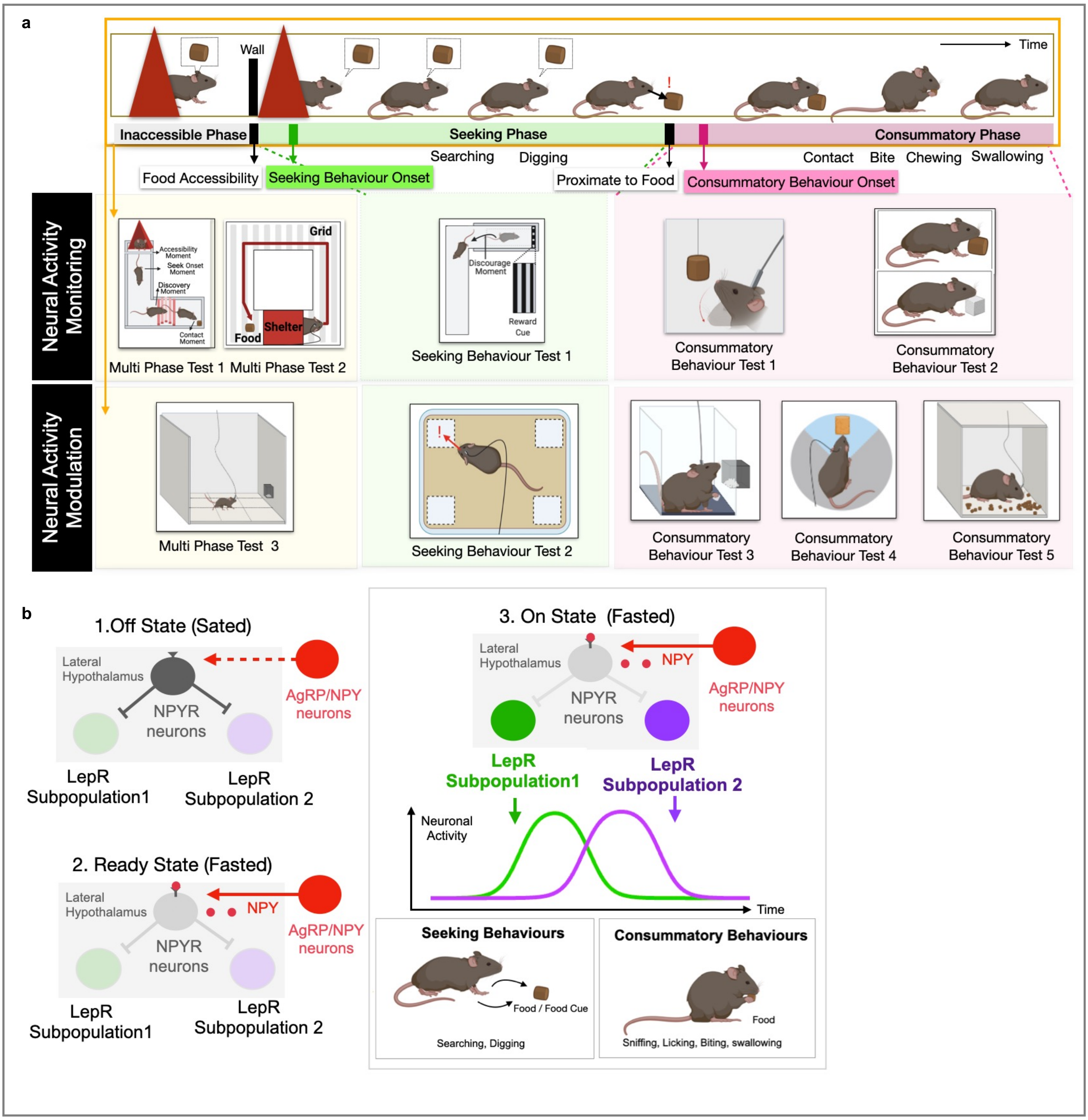

Supplementary Figure. 9 | Experimental paradigms for seeking and consummatory behaviours.

a, Comprehensive summary of behavioural experiments to observe specific phases of feeding behaviours

b, Graphical summary of mechanism between hunger-gated permissive role of NPY and two distinct population of seeking and consummatory LH^{LepR} neurons. The schematics in a and b were created using BioRender.

	Figure	Mouse	Virus	Mouse	Behavior test	Specific Figure	Statistics	N	Trials	Time bin	p-value
Figure 1	Figure 1C,E,G,H,I	Vgat	GCaMP6s	Vgat85, Vgat88, Vgat96,Vgat97, Vgat98,Vgat99	Multi-phase test 2, Consummatory behaviour test 1, Consummatory behaviour test 2, 1 non-food context test	-	-	6	-	-	-
	Figure 1D,F,J, K,L	LepR	GCaMP6s	LepR54-2, LepR64-1, LepR64-2, LepR65-1		-	-	4	-	-	-
Figure 2	Figure 2C-G	LepR	GCaMP6s	LepR20-4, LepR26, LepR27, LepR38, LepR40-1	Consummatory behaviour test 1 (8cm)	Figure 2F	2-Way ANOVA	5	22	-	2.68E-09
	Figure 2H-O	LepR	GCaMP6s	LepR20-4, LepR26, LepR38, LepR40-1	Multi-phase test 2	Figure 2K	2-Way ANOVA	4	67	-	2.19E-11
						Figure 2O	two-tailed one-sample t-test	4	66	-	1.77E-20
	Figure 2P-U	LepR	GCaMP6s	LepR20-4, LepR26, LepR38, LepR40-1	Multi-phase test 1	Figure 2T	paired t-test	4	-	-	0.208793
						Figure 2U	paired t-test	4	-	-	0.029257
Figure 3	Figure 5A-M	LepR	GCaMP6s	LepR54-2, LepR64-1, LepR64-2, LepR65-1	Multi-phase test 2	-	-	4	-	-	-
Figure 4	Figure 4C-G	LepR	ChR2	LepR 127-4, LepR 128-1, LepR 128-2, LepR 128-4	Multi-phase test 3	Figure 4D	paired t-test	4	-	pre,laser	0.6042
							paired t-test			pre,post	0.5551
							paired t-test			laser,post	0.6562
						Figure 4E	paired t-test	4		pre,laser	0.9291
							paired t-test			pre,post	0.4742
							paired t-test			laser,post	0.3307
						Figure 4F	paired t-test	4		pre,laser	0.2657
							paired t-test			pre,post	0.7745
							paired t-test			laser,post	0.2823
						Figure 4G	paired t-test	4		pre,laser	0.6853
							paired t-test			pre,post	0.1653
							paired t-test			laser,post	0.1515
	Figure 4H-N	LepR	ChR2	LepR43-4, LepR51-1, LepR51-5, LepR53-3, LepR53-4, LepR53-6 LepR53-1	Seeking behaviour test 2	Figure 4K	paired t-test	7	-	pre,laser	0.020644
							paired t-test			pre,post	0.103837
							paired t-test			laser,post	0.16916
						Figure 4L	paired t-test	7		pre,laser	0.041032
							paired t-test			pre,post	0.01432
							paired t-test			laser,post	0.003727
						Figure 4M	paired t-test	7		pre,laser	0.002864
							paired t-test			pre,post	0.001877
							paired t-test			laser,post	0.000183
						Figure 4N	paired t-test	7		pre,laser	0.040136
							paired t-test			pre,post	0.282722
							paired t-test			laser,post	0.073217
	Figure 4O-S	LepR	ChR2	LepR 91-1, LepR90-3, LepR91-3, LepR109-4, LepR 111-2	Consummatory behaviour test 3	Figure 4Q	paired t-test	5	-	pre,laser	0.0071
							paired t-test	5		pre,post	0.7142
							paired t-test	5		laser,post	0.0105
						Figure 4R	paired t-test	5		pre,laser	0.0105
							paired t-test	5		pre,post	0.8404
							paired t-test	5		laser,post	0.0056
						Figure	paired t-test	5		pre,laser	0.0153

						4S	paired t-test	5		pre,post	0.9738
							paired t-test	5		laser,post	0.0177
Figure 5	Figure 4A-J	LepR	NpHR	LepR 63, LepR67-1, LepR67-2, LepR67-3, LepR67-5, LepR 118-2, LepR 119-4, LepR 120-3	Consummatory behaviour test 5	Figure 5D	paired t-test	8	-	off(0-2),on(2-4)	0.208933855
										on(2-4),off(4-6)	0.006050549
										off(4-6),on(6-8)	0.00078894
										on(6-8),off(8-10)	0.14801064
										off(8-10),on(10-12)	0.133411626
										on(10-12),off(12-14)	0.000936386
										off(12-14),on(14-16)	0.029581051
										on(14-16), off(16-18)	0.019030718
										off(16-18),on(18-20)	0.423020949
						Figure 5E	paired t-test	8		off,on	7.14E-05
						Figure 5F	paired t-test	8		off,on	0.000995496
						Figure 5G	paired t-test	8		off,on	0.937595033
Figure 6	Figure 6A-J	LepR	GCaMP6s	-	Brain slice calcium imaging	Figure 6H-left	paired t-test	6/8 slices	65/69	before NPY application, After NPY application	0.0002
						Figure 6H-right	unpaired t-test	6/8 slices	65/69	NPY, Antagonist + NPY	0.014947
						Figure 6I-left	paired t-test	6/8 slices	65/69	before NPY application, After NPY application	0.0195
						Figure 6I-right	Unpaired t-test	6/8 slices	65/69	NPY, Antagonist + NPY	0.0029843
						Figure 6J	Unpaired t-test	6/8 slices		NPY, Anta + NPY	0.0133
	Figure 6K-N	LepR - tomato	-	-	Brain slice whole-cell recording	Figure 6M	2-Way ANOVA	8/6 cells	-	ACSF,NPY	0.0005
						Figure 6N	2-Way ANOVA	8/6 cells	-	ACSF,NPY	0.2209
						Figure 6O-left	paired t-test	8cells	-	ACSF,NPY	0.9194
						Figure 6O-right	paired t-test	6cells	-	ACSF,NPY	0.0431
						Figure 6P-left	paired t-test	8cells	-	ACSF,NPY	0.0552
						Figure 6P-right	paired t-test	6cells	-	ACSF,NPY	0.0862
Supplement Figure											
Supplementary figure 1	Supplementary Fig 1A-K	LepR - tomato	-	LepR-toma Old, LepR-toma 1, LepR toma2	2D imaging	-	-	3	-	-	-
Supplementary figure 2	Supplementary Fig 2A-E	LepR	GCaMP6s	LepR54-2, LepR64-1, LepR64-2, LepR65-1	Water test	-	-	4	-	-	-
	Supplementary Fig 2F-J	LepR	GCaMP6s	LepR20-4,LepR26,LepR38, LepR40-1	Consummatory behaviour test 2	Sup Fig 2I-left	2-Way ANOVA	4	32	-	0.762194
						Sup Fig 2I-right	2-Way ANOVA	4	32	-	2.74E-14
	Supplementary Fig 2K	Vgat, LepR		Vgat85, Vgat88, Vgat96,Vgat97, Vgat98,Vgat99 LepR54-2, LepR64-1, LepR64-2, LepR65-1	Multi-phase test 2, Consummatory behaviour test 1, Consummatory behaviour test 2, 1 non-food context test	Sup Fig 2L	Chi-Square Statistic of cross-tabulation table	6,4	218, 48	GABA,LepR	5.98E-17
Supplementary figure 3	Supplementary Fig 3A-E	LepR	GCaMP6s	LepR 38	Seeking behaviour test 1	Sup Fig 3D	2-Way ANOVA	1	5	-	0.009104
	Supplementary Fig 3F-J	LepR	GCaMP6s	LepR20-4,LepR26,LepR27, LepR38, LepR40-1	Consummatory behaviour test 1 (11cm)	Sup Fig 3I	2-Way ANOVA	5	37	-	0.000013

Supplementary figure 4	Supplementary Fig 4A-B	LepR	GCaMP6s	LepR54-2,LepR64-1,LepR64-2, LepR65-1	-	-	-	4	-	-	-
Supplementary figure 5	Supplementary Fig 5A-E	LepR	EYFP	EYFP115-4, EYFP120-1,EYFP121-2, EYFP121-3, EYFP 121-4	Multi-phase test 3	Sup Fig 5B	paired t-test	5	-	pre,laser	0.2052
							paired t-test	5	-	pre,post	0.04910
							paired t-test	5	-	laser,post	0.9245
						Sup Fig 5C	paired t-test	5	-	pre,laser	0.4405
							paired t-test	5	-	pre,post	0.9343
							paired t-test	5	-	laser,post	0.3372
						Sup Fig 5D	paired t-test	5	-	pre,laser	0.1419
							paired t-test	5	-	pre,post	0.7780
							paired t-test	5	-	laser,post	0.1813
						Sup Fig 5E	paired t-test	5	-	pre,laser	0.6506
							paired t-test	5	-	pre,post	0.2665
							paired t-test	5	-	laser,post	0.1903
	Supplementary Fig 5F-J	LepR	NpHR	NPHR 116-2, NPHR 118-2, NPHR 119-4, NPHR 120-3	Multi-phase test 3	Sup Fig 5G	paired t-test	4	-	pre,laser	0.6614
							paired t-test	4		pre,post	0.0324
							paired t-test	4		laser,post	0.5702
						Sup Fig 5H	paired t-test	4		pre,laser	0.7493
							paired t-test	4		pre,post	0.5474
							paired t-test	4		laser,post	0.4420
						Sup Fig 5I	paired t-test	4	-	pre,laser	0.1419
							paired t-test	4	-	pre,post	0.7780
							paired t-test	4	-	laser,post	0.1813
						Sup Fig 5J	paired t-test	4	-	pre,laser	0.6506
							paired t-test	4	-	pre,post	0.2665
							paired t-test	4	-	laser,post	0.1903
	Supplementary Fig 5K-N	LepR	EYFP	EYFP115-4, EYFP120-1, EYFP121-2, EYFP121-3, EYFP 121-4	Seeking behaviour test 2	Sup Fig 5L	paired t-test	5	-	pre,laser	0.3192
							paired t-test	5	-	pre,post	0.8620
							paired t-test	5	-	laser,post	0.2577
						Sup Fig 5M	paired t-test	5	-	pre,laser	0.7590
							paired t-test	5	-	pre,post	0.0751
							paired t-test	5	-	laser,post	0.0671
						Sup Fig 5N	paired t-test	5	-	pre,laser	0.0917
							paired t-test	5	-	pre,post	0.0029
							paired t-test	5	-	laser,post	0.0183
	Supplementary Fig 5P-R	LepR	NpHR	NPHR 116-2, NPHR 118-2, NPHR 119-4, NPHR 120-3	Seeking behaviour test 2	Sup Fig 5P	paired t-test	4	-	off,on	0.0090
						Sup Fig 5Q	paired t-test	4	-	off,on	0.1201
						Sup Fig 5R	paired t-test	4	-	off,on	0.3082
	Supplementary Fig 5T-W	LepR	EYFP	EYFP 90-1 EYFP115-4, EYFP120-1, EYFP121-2, EYFP121-3, EYFP 121-4	Consummatory behaviour test 4	Sup Fig 5T	paired t-test	5	-	pre,laser	0.0900
							paired t-test	5	-	pre,post	0.1552
							paired t-test	5	-	laser,post	0.4760
						Sup Fig 5U	paired t-test	5	-	pre,laser	0.3615
							paired t-test	5	-	pre,post	0.2370
							paired t-test	5	-	laser,post	0.2330

						Sup Fig 5V	paired t-test	5	-	pre,laser	0.2698
							paired t-test	5	-	pre,post	0.2624
							paired t-test	5	-	laser,post	0.8452
						Sup Fig 5W	two sample Kolmogorov -Smirnov test	5	-	CHR2,EYFP	0.0020
Supplementary figure 6	Supplementary Fig 6A-D	LepR	ChR2	LepR43-4,LepR51-1,LepR51-5, LepR53-3,LepR53-4,LepR53-6	Consummatory behaviour test 4	Sup Fig 6D	paired t-test	6	-	pre,laser	0.0014
							paired t-test	6	-	pre,post	0.0666
							paired t-test	6	-	laser,post	0.0013
	Supplementary Fig 6E-I	LepR	ChR2	LepR 71-1, LepR 73-1, LepR 73-2, LepR 74-2	Consummatory behaviour test 4	Sup Fig 6I	paired t-test	4	-	No laser, laser	0.0038
Supplementary figure 7	Supplementary Fig 7A-H	LepR	EYFP	EYFP 90-1 EYFP115-4, EYFP120-1, EYFP121-2, EYFP121-3, EYFP 121-4	Consummatory behaviour test 5	Sup Fig 7B	paired t-test	6	-	off(0-2),on(2-4)	0.3327
								6	-	on(2-4),off(4-6)	0.7287
								6	-	off(4-6),on(6-8)	0.1720
								6	-	on(6-8),off(8-10)	0.6300
								6	-	off(8-10),on(10-12)	0.0475
								6	-	on(10-12),off(12-14)	0.0560
								6	-	off(12-14),on(14-16)	0.2271
								6	-	on(14-16), off(16-18)	0.3010
								6	-	off(16-18),on(18-20)	0.3792
						Sup Fig 7C	paired t-test	6	-	off, on	0.0502
						Sup Fig 7D	paired t-test	6	-	off, on	0.1208
						Sup Fig 7E	paired t-test	6	-	off, on	0.2037
Supplementary figure 8	Supplementary Fig 8A-D	LepR	GCaMP6		Brain slice calcium imaging	Sup Fig 8C	paired t-test	6slices	-	ACSF, Leptin	0.2154
			GCaMP6		Brain slice calcium imaging	Sup Fig 8D	Unpaired t-test	6/6 slices	-	Leptin, NPY	0.0012

Supplementary Table. 1 Summary of statistical analyses.
