# Supplementary material for: Lateral hypothalamic leptin receptor neurons drive hunger-gated food-seeking and consummatory behaviours in male mice
Source: Nat Commun. 2023 Mar 17;14:1486. doi: 10.1038/s41467-023-37044-4 (PMC10023672; doi:10.1038/s41467-023-37044-4)
Supplement: Supplementary file 2 — Description of Additional Supplementary Files [file 41467_2023_37044_MOESM2_ESM.pdf]

Description of Additional Supplementary Files

File Name: Supplementary Movie 1
Description: LH^{LepR} 3D mapping, related to Supplementary Figure 1.

File Name: Supplementary Movie 2
Description: LH^{LepR} neural activity increases during food-seeking and consummatory behaviour. The grey line indicates LH^{LepR} neural activity during behavioural tests. The black line indicates neural activity fitted to the 9th order polynomial. 00:00-01:04 shows consummatory behaviour test 1 (obtainable). 01:04-03:04 shows multi-phase test 1 before and after conditioning. 03:04-03:18 shows multi-phase test 2. The yellow line indicates the third derivative of neural activity (jolt) used to calculate neural onset. Gray dotted line is showed at the moment of maximum value of jolt. 03:18-03:44 shows consummatory behaviour test 1 (unobtainable). LH^{LepR} neural activity starts decreasing at the termination of consummatory behaviour.

File Name: Supplementary Movie 3
Description: Activation of LH^{LepR} neurons drives food-seeking and consummatory behaviours. 00:00-01:14 shows the seeking behaviour test 2. The yellow box indicates the food zone. 01:14-02:01 shows the consummatory behaviour test 3. 02:01-02:21 shows the consummatory behaviour test 4. 02:21-06:24 shows the consummatory behaviour test 5. The black box indicates the duration of behaviour. If the mouse completed consummatory behaviours, a blue line was displayed at the start of the black box. If the mouse did not complete consummatory behaviours, a red line was displayed at the start of the black box.

File Name: Supplementary Movie 4
Description: Identification of consummatory behaviours by DeepLabCut.

File Name: Supplementary Movie 5
Description: Two distinct populations of LH^{LepR} neurons encode drive for food-seeking and consummatory behaviours. The upper right area shows activated LH^{LepR} neurons. Darker colour represents higher cell activity. 00:04-00:27 shows a food trial (seeking with consummatory behaviours). 00:27-00:50 shows a no-food trial (seeking without consummatory behaviours).
