# Supplementary material for: Lateral hypothalamic leptin receptor neurons drive hunger-gated food-seeking and consummatory behaviours in male mice
Source: Nat Commun. 2023 Mar 17;14:1486. doi: 10.1038/s41467-023-37044-4 (PMC10023672; doi:10.1038/s41467-023-37044-4)
Supplement: Supplementary file 8 — Reporting Summary [file 41467_2023_37044_MOESM8_ESM.pdf]

nature portfolio

Corresponding author(s): Hyung Jin Choi

Last updated by author(s): Feb 10, 2023

Reporting Summary

Nature Portfolio wishes to improve the reproducibility of the work that we publish. This form provides structure for consistency and transparency in reporting. For further information on Nature Portfolio policies, see our Editorial Policies and the Editorial Policy Checklist.

Statistics

For all statistical analyses, confirm that the following items are present in the figure legend, table legend, main text, or Methods section.

n/a	Confirmed	
☐	☒	The exact sample size (*n*) for each experimental group/condition, given as a discrete number and unit of measurement
☐	☒	A statement on whether measurements were taken from distinct samples or whether the same sample was measured repeatedly
☐	☒	The statistical test(s) used AND whether they are one- or two-sided<br>*Only common tests should be described solely by name; describe more complex techniques in the Methods section.*
☒	☐	A description of all covariates tested
☐	☒	A description of any assumptions or corrections, such as tests of normality and adjustment for multiple comparisons
☐	☒	A full description of the statistical parameters including central tendency (e.g. means) or other basic estimates (e.g. regression coefficient) AND variation (e.g. standard deviation) or associated estimates of uncertainty (e.g. confidence intervals)
☐	☒	For null hypothesis testing, the test statistic (e.g. *F*, *t*, *r*) with confidence intervals, effect sizes, degrees of freedom and *P* value noted<br>*Give P values as exact values whenever suitable.*
☒	☐	For Bayesian analysis, information on the choice of priors and Markov chain Monte Carlo settings
☒	☐	For hierarchical and complex designs, identification of the appropriate level for tests and full reporting of outcomes
☒	☐	Estimates of effect sizes (e.g. Cohen's *d*, Pearson's *r*), indicating how they were calculated

Our web collection on statistics for biologists contains articles on many of the points above.

Software and code

Policy information about availability of computer code

Data collection	Doric Neuroscience Studio (v5),Inscopix nVoke system,Noldus Ethovision(v14), Olympus FV31S-SW (v2.3.2.169), SPIM (RaVision), VisiView software (1.1.1)
Data analysis	MathWorks MATLAB (R2022b), IMARIS (9.5), IBM SPSS 25.0

For manuscripts utilizing custom algorithms or software that are central to the research but not yet described in published literature, software must be made available to editors and reviewers. We strongly encourage code deposition in a community repository (e.g. GitHub). See the Nature Portfolio guidelines for submitting code & software for further information.

Data

Policy information about availability of data

All manuscripts must include a data availability statement. This statement should provide the following information, where applicable:

- Accession codes, unique identifiers, or web links for publicly available datasets
- A description of any restrictions on data availability
- For clinical datasets or third party data, please ensure that the statement adheres to our policy

The neural and behavioral data used to generate the figure in this study has been deposited in Figshare repository under accession code https://doi.org/10.6084/m9.figshare.22058123.v2.

Human research participants

Policy information about studies involving human research participants and Sex and Gender in Research.

Reporting on sex and gender	N/A
Population characteristics	N/A
Recruitment	N/A
Ethics oversight	N/A

Note that full information on the approval of the study protocol must also be provided in the manuscript.

Field-specific reporting

Please select the one below that is the best fit for your research. If you are not sure, read the appropriate sections before making your selection.

☒ Life sciences ☐ Behavioural & social sciences ☐ Ecological, evolutionary & environmental sciences

For a reference copy of the document with all sections, see nature.com/documents/nr-reporting-summary-flat.pdf

Life sciences study design

All studies must disclose on these points even when the disclosure is negative.

Sample size	Sample sizes were based on previous studies in the field of behavioral neuroscience.The list of references is as follows:<br>-2015,Cell, Jennings et al. (Visualizing Hypothalamic Network Dynamics for Appetitive and Consummatory Behaviors)<br>-2017, Nature, Livneh et al. (Homeostatic circuits selectively gate food cue responses in insular cortex)<br>-2018, Nature Neuroscience, Giardino et al. (Parallel circuits from the bed nuclei of stria terminalis to the lateral hypothalamus drive opposing emotional states)<br>-2021, Nature Neuroscience, Sharpe et al.(Past experience shapes the neural circuits recruited for future learning)
Data exclusions	Viral expressionand implant placement were verified by histology before the data were included in the analysis.
Replication	To ensure that our experimental findings can be reliably replicated, each experiment was repeated at least three times by multiple experimenters. All behavioral and recording experiments were conducted with 3-10 times on separate cohorts. All attempts for replication were successful.
Randomization	For behavioral experiments, mice were randomly assigned to experimental and control groups before surgery and experiments.
Blinding	For optogenetics and brain slice study, the investigators were blinded to group allocation during data collection. For fiber photometry, and micro-endoscope experiments, the investigators were not blinded. However, data analyses were automatically performed using MATLAB with the same scripts run for each experimental group.

Reporting for specific materials, systems and methods

We require information from authors about some types of materials, experimental systems and methods used in many studies. Here, indicate whether each material, system or method listed is relevant to your study. If you are not sure if a list item applies to your research, read the appropriate section before selecting a response.

Materials & experimental systems

n/a	Involved in the study	
☒	☐	Antibodies
☒	☐	Eukaryotic cell lines
☒	☐	Palaeontology and archaeology
☐	☒	Animals and other organisms
☒	☐	Clinical data
☒	☐	Dual use research of concern

Methods

n/a	Involved in the study	
☒	☐	ChIP-seq
☒	☐	Flow cytometry
☒	☐	MRI-based neuroimaging

Animals and other research organisms

Policy information about studies involving animals; ARRIVE guidelines recommended for reporting animal research, and Sex and Gender in Research

Laboratory animals	Adult male mice (at least 8-weeks-old) of the following strains were used: LepR-Cre (JAX stock no.008320), Ai-14 Td-Tomato (JAX stock no.007914), Vgat-Cre (JAX stock no. 028862)
Wild animals	No wild animals were used.
Reporting on sex	All mice using the experiments were male.
Field-collected samples	No field-collected samples were used.
Ethics oversight	Experimental procedures were performed in compliance with the Guide for the Care and Use of Laboratory Animals from the Seoul National University, and approved by the Seoul National University Institutional Animal Care and Use Committee.

Note that full information on the approval of the study protocol must also be provided in the manuscript.
